# Independent wing reductions and losses among stick and leaf insects (Phasmatodea), supported by new Cretaceous fossils in amber

**DOI:** 10.1186/s12915-023-01720-0

**Published:** 2023-10-09

**Authors:** Hongru Yang, Michael S. Engel, Chungkun Shih, Fan Song, Yisheng Zhao, Dong Ren, Taiping Gao

**Affiliations:** 1 College of Life Sciences, Capital Normal University, Beijing, 100048 China; 2 Division of Invertebrate Zoology, American Museum of Natural History, New York, NY 10024 USA; 3 Department of Paleobiology, National Museum of Natural History, Smithsonian Institution, Washington, DC 20013-7012 USA; 4 Department of Entomology MOA Key Lab of Pest Monitoring and Green Management, College of Plant Protection, China Agricultural University, Beijing, 100193 China

**Keywords:** Holophasmatodea, Timematodea, Phylogeny, Wing evolution, Biogeographic occurrence, Anti-predation strategies

## Abstract

**Background:**

Phasmatodea (stick and leaf insects) play a central role on the debate regarding wing reduction and loss, and its wings are putative reacquisition from secondarily wingless ancestors based solely on extant species. A pivotal taxon in this respect is the species-poor Timematodea, consisting of approximately 21 wingless extant species, which form the sister group of all remaining winged or wingless stick and leaf insects, the Euphasmatodea.

**Results:**

Herein, the new fossils of Timematodea from mid-Cretaceous Kachin amber are reported, with winged and wingless species co-occurring. The palaeogeographic distributions of all fossils of Holophasmatodea are summarized, showing their wide paleo-distributions. The phylogenetic analysis based on morphological characters confirms the earliest-diverging lineage of winged *Breviala cretacea* gen. et sp. nov. in Timematodea, and the possible relationships among all families of Holophasmatodea. These are critical for the reconstruction of patterns of wing evolution in early Phasmatodea.

**Conclusions:**

The new fossils suggest that Timematodea once had wings, at least during the mid-Cretaceous. The palaeogeographic occurrences imply that Timematodea probably have been widely distributed since at least the Jurassic. The phylogenetic analysis with the ancestral-state reconstruction of wings indicates that the common ancestors of Holophasmatodea were winged, the reductions and losses of wings among Timematodea and Euphasmatodea have occurred independently since at least the Cretaceous, and the reduction or loss of the forewing earlier than the hind wings.

**Supplementary Information:**

The online version contains supplementary material available at 10.1186/s12915-023-01720-0.

## Background

Wings are iconic structures among insects, with more than 95% of all insects capable of flight, and these are credited along with a plethora of other specializations for the considerable success of Hexapoda [1–3]. Yet, despite the monophyletic origin of wings and considerable success of flying insects, flight and wings have been lost and rendered vestigial or absent an innumerable number of times in the last 325 million years [1]. One group in particular has been the focus of considerable debate regarding wing reduction and loss, and its putative reacquisition from secondarily wingless ancestors [4–7]. The insect order Phasmatodea, the stick and leaf insects, exhibit a complex history and pattern of wing trait evolution. Some molecular studies have posited that ancestors of crown-Phasmatodea were wingless and that wings were “re-evolved” subsequently during their evolution, even multiple times perhaps [4–6]. Regardless of character polarity, such numerous transitions between winged and wingless are likely the result of the deactivation of wing expression, while the genetic architecture for the wing remains present, albeit dormant and ready to be re-expressed [7]. In these studies, based solely on extant taxa, character information on wings from extinct Phasmatodea, and particularly from stem groups comprising the broader clade Holophasmatodea, have largely been ignored, despite the assertion that fully developed wings constitute a groundplan feature for stick insects [1, 8], contrary to ancestral-state reconstructions based solely on living taxa [4, 5]. Regardless, the history of wing evolution and loss among Phasmatodea is complex and involves many reversals, although the polarity of these changes remains controversial.

In insects wings first evolved to as a means of accessing food, aiding dispersal, and escaping predators [1]. Subsequently, wings have been augmented into varied forms, and to serve many functions, these are frequently associated with the specific mechanics of certain kinds of flight, although cooption for other purposes is common, such as communication, mating, defense, or camouflage [2]. As noted, the reduction and loss of wings is frequent in extant stick and leaf insects. There are also fully-winged species capable of good flight, but the wings of many relatively macropterous lineages are more often used to control free-fall descents from tree canopies [9–12]. Some brachypterous lineages have also evolved non-aerodynamic functions for wings, such as aposematic coloration or a stridulation capability [5]. Some species can produce disruptive sounds by rubbing the tegmina (forewing) against the remigium of the hind wing [13], and can also produce a startling visual display by suddenly raising the tegmina and flashing bright colors or patterns on the relatively large hind wings [14]. The wings of leaf insects (Phylliidae) simulate the color and shape of a leaf, while the wing venation closely resembles leaf veins. Few species with wing remnants are present in the otherwise apterous lineages, such as *Agathemera*, but a function remains unknown for those that do retain vestiges [15]. Regardless, a stark difference can be observed between the wings of extinct and extant species of Phasmatodea, at least in terms of their overall size, development, and specializations of venation. Most fossil stick insects have two pairs of fully developed wings, with few having lost or reduced wings, and most of the latter are from more derived extant clades and are often comparatively young in age [16–18]. Many extant Phasmatodea have either reduced wings or lost them outright, although likewise, many notable exceptions do exist, reflective of the complex mosaic of phasmatodean evolution.

Currently, definitive Phasmatodea s.l. comprise the extinct Susumaniidae and Pterophasmatidae [16, 17], along with the extant sister clades Timematodea and Euphasmatodea [18–20]. The more ancient, extinct families of Permophasmatidae, Prochresmodidae, Xiphopteridae, Aeroplanidae, and Aerophasmatidae are considered to be stem-groups of Phasmatodea (as the larger group Holophasmatodea), although definitive characters clarifying their phylogenetic positions remain somewhat elusive [21]. The species-poor Timematodea comprise only 21 extant species within a single genus, *Timema*, and are supported as the sister group of all other extant Phasmatodea, the latter of which comprise the Euphasmatodea [4–6, 18–20, 22–25]. The fact that *Timema* are completely wingless and occupy such an early diverging position relative to Euphasmatodea certainly biases ancestral-state reconstructions based solely on extant taxa alone. It is therefore of considerable relevance that newly discovered fossils of Timematodea from the mid-Cretaceous of Asia reveal species retaining wings, with fossils of winged and wingless species alike co-occurring during the period. Taken together, these fossils reveal the loss of wings in Timematodea is itself a rather complex story and highlights the more extensive pattern of repeated wing losses among Phasmatodea as well as the critical importance of utilizing fossils rather than relying solely on extant groups for ancestral-state reconstructions.

## Results

### Systematic palaeontology

Order Phasmatodea Jacobson & Bianchi, 1902.

Suborder Timematodea Kevan, 1977.

Family Timematidae Caudell, 1903.

**Fig. 1:**
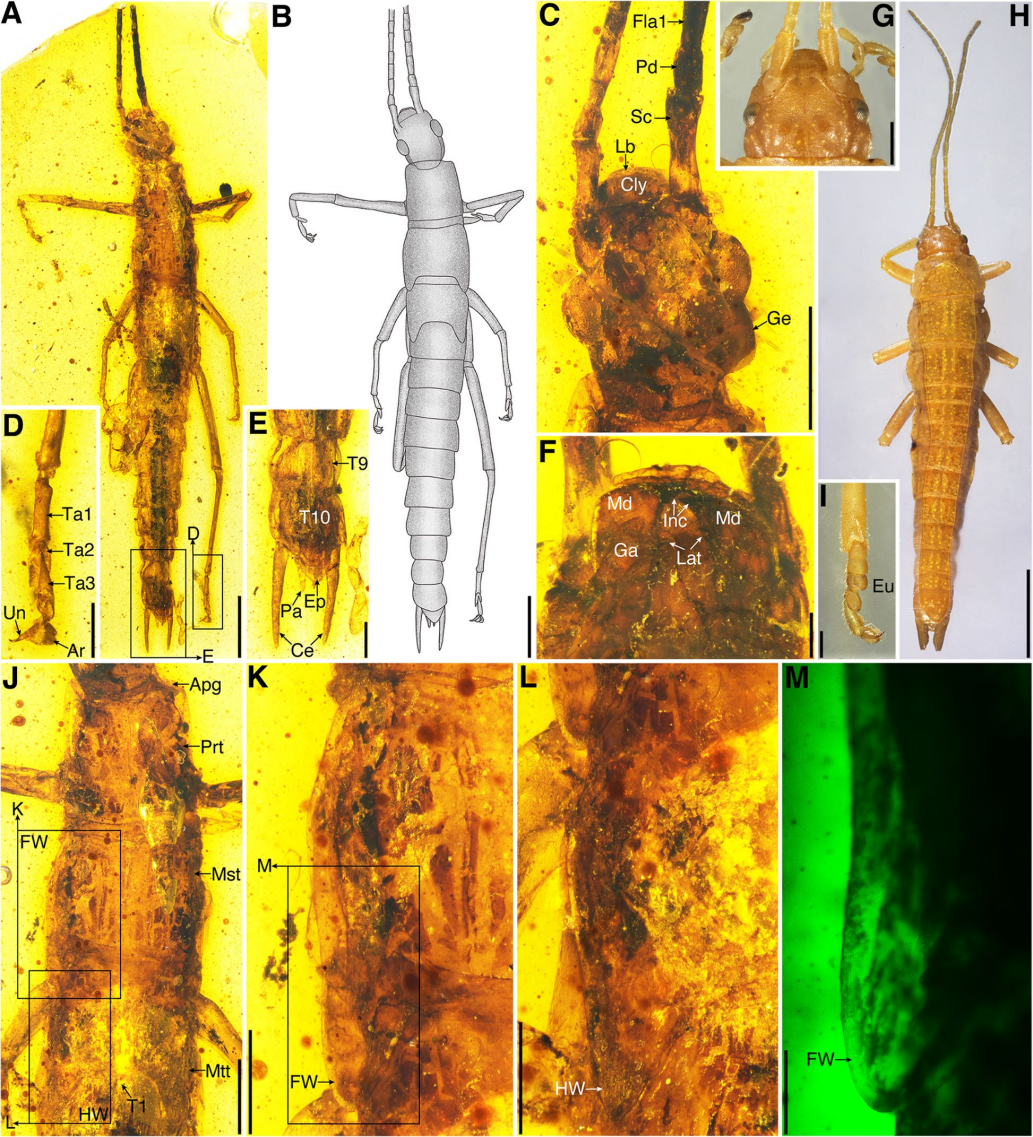
Holotype of *Breviala cretacea* (No. CNU-PHA-MA2016017) (**A**–**F**, **J**–**M**) and *Timema chumash* (**G**–**I)**. **A** Habitus. **B** Line drawing. **C** Head in dorsal view. **D** Metatarsus. **E** Male genitalia. **F** Head in ventral view. **G** Head in dorsal view. **H** Habitus. **I** Protarsus. **J** Thorax. **K** Forewing bud. **L** Hind wing bud. **M** Forewing bud. Scale bars: **A**, **B** 2 mm; **C**, **G**, **J** 1 mm; **H** 5 mm; **D**, **E**, **I**, **K**, **L** 0.5 mm; **F**, **M** 0.2 mm

**Fig. 2:**
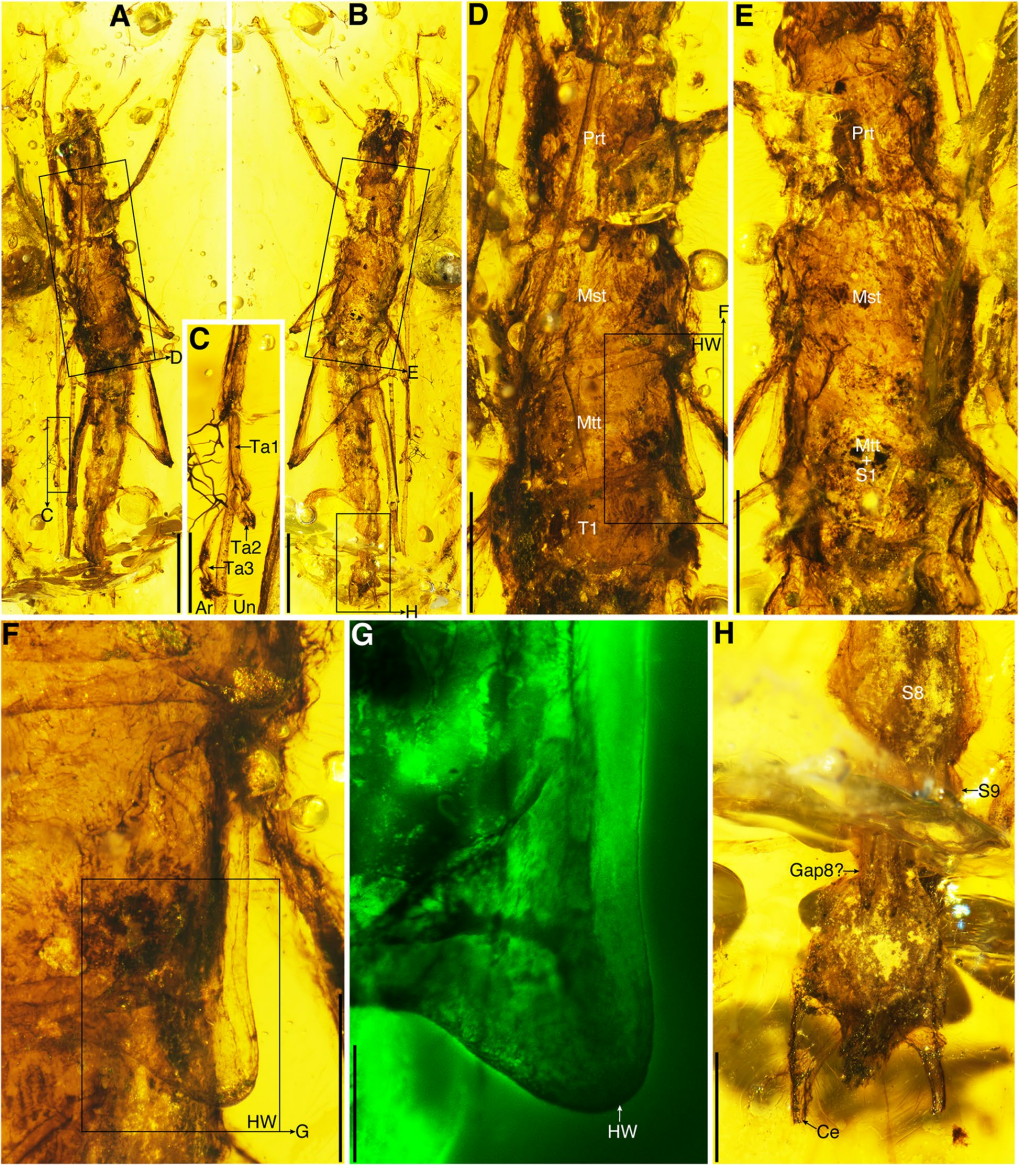
Paratype of *Breviala cretacea* (No. CNU-PHA-MA2016018). **A** Habitus in dorsal view. **B** Habitus in ventral view. **C** Mesotarsus. **D** Thorax in dorsal view. **E** Thorax in ventral view. **F** Metathorax and hind wing. **G** Hind wing bud. **H** Female genitalia. Scale bars: **A**, **B** 2 mm; **C**, **F**, **H** 0.5 mm; **D**, **E** 1 mm; **G** 0.2 mm

***Breviala *Yang, Engel, Shih & Gao gen. nov.** (Figs. 1 and 2).

ZooBank LSID: urn:lsid:zoobank.org:act:B9792FD4-3DA0-4B69-95E5-5544B98CD951.

**Type species.** *Breviala cretacea* Yang, Engel, Shih & Gao sp. nov.

**Etymology.** The new generic name is a combination of the Latin *brevis* (meaning, “short”) and *āla* (meaning, “wing”) (gender of the name is feminine).

**Diagnosis.** Head ovoid, longer than width; compound eye ovoid, about 1/3 as long as head, maxilla with two lacinial teeth; the length of pro- and mesothorax almost equal, metathorax short and similar length with abdominal segment I, wing buds of meso- and metathorax present, thoracic terga surrounded by extensive membrane; legs slender and shorter than abdomen; epiproct large, triangular; cercus strongly elongate, gradually tapering toward the apex.

***Breviala cretacea*** **Yang, Engel, Shih & Gao sp. nov.**

ZooBank LSID: urn:lsid:zoobank.org:act:E79F1468-5A4C-4890-8C6A-83B2F3BDB194.

**Etymology.** The specific epithet is from the Latin adjective *crētācea*, referencing the Cretaceous.

**Diagnosis.** As for the genus (vide supra).

**Materials.** Holotype, a 3rd or 4th instar male, No. CNU-PHA-MA2016017; Paratype, a 3rd or 4th instar female, No. CNU-PHA-MA2016018.

**Fig. 3:**
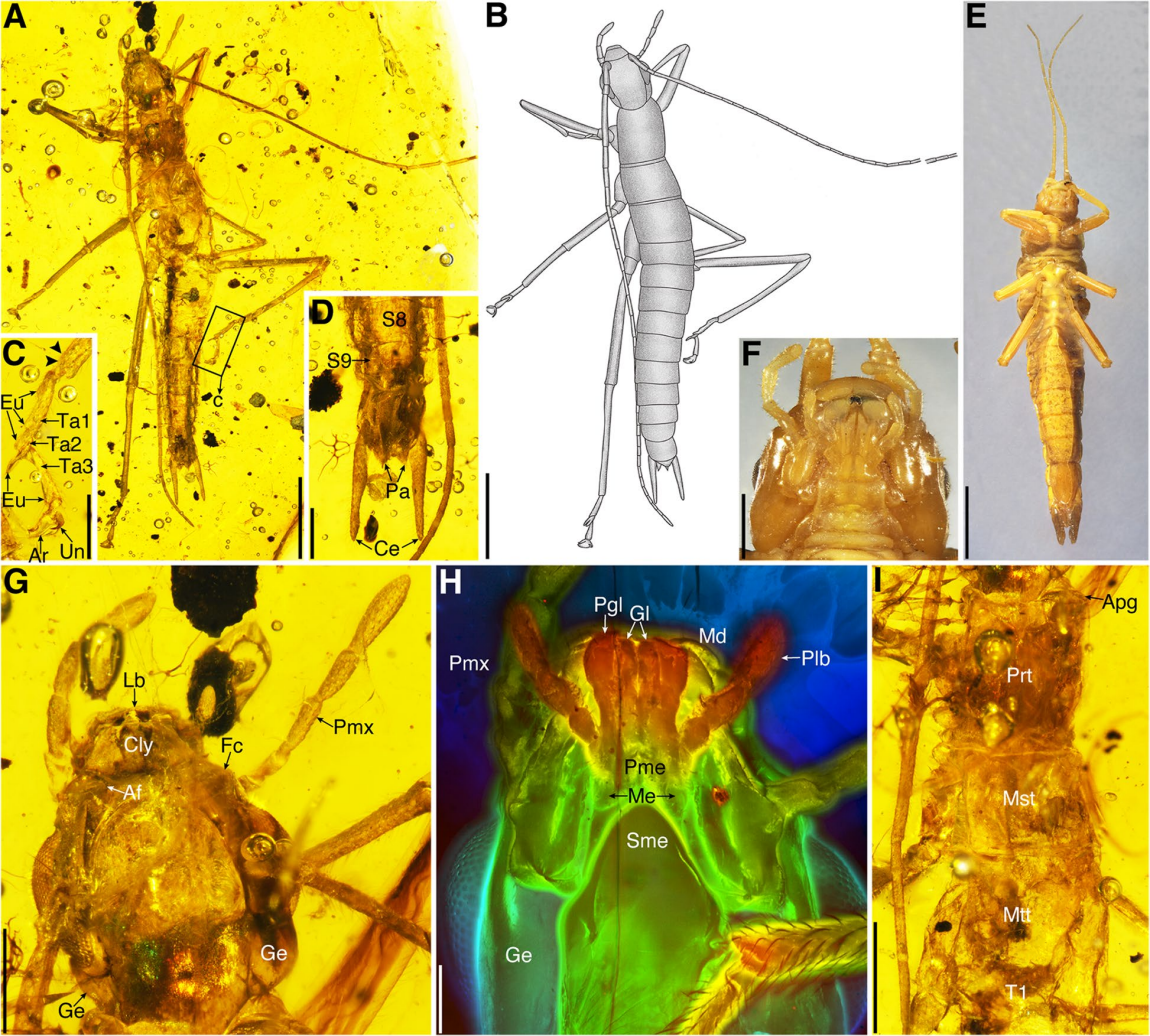
Holotype of *Electroclavella genuine* (No. CNU-PHA-MA2016019) (**A**–**D**, **G**–**I**) and *Timema chumash* (**E**, **F)**. **A** Habitus in dorsal view. **B** Line drawing. **C** Metatarsus. **D** Male genitalia in ventral view.** E** Habitus in ventral view. **F** Mouthparts.** G** Head in dorsal view. **H** Mouthparts. **I** Thorax in dorsal view. Scale bars: **A**, **B** 2 mm; **E** 5 mm; **C**, **D**, **G** 0.5 mm; **F**, **I** 1 mm; **H** 0.2 mm

***Electroclavella *Yang, Engel, Shih & Gao gen. nov.** (Fig. 3, Additional file 1: Fig. S1).

ZooBank LSID: urn:lsid:zoobank.org:act:BC798F05-E038-48F1-8941-25B8123080E6.

**Type species.** *Electroclavella genuina* Yang, Engel, Shih & Gao sp. nov.

**Etymology.** The new generic name is a combination of the Latin words *ēlectrum *(meaning, “amber”), *clāva* (meaning, “club”), and the suffix –*ella* (indicating a diminutive) (gender of the name is feminine).

**Diagnosis.** Head globular, slightly raised posteriorly; compound eye nephroid, about 1/2 as long as head, glossa and paraglossa long and narrow, mentum with two triangular sclerites, submentum cuspidal apically; prothorax longer than meso- and metathorax, meso- and metathorax similar length and longer than abdominal segment I, wing buds absent, thoracic terga surrounded by narrow membrane; leg slender and longer than abdomen; epiproct small, triangular; cercus strongly elongate, inconsistent width.

***Electroclavella genuina*** **Yang, Engel, Shih & Gao sp. nov.**

ZooBank LSID: urn:lsid:zoobank.org:act:25225173–7271-41F1-8483-678A41B3B53C.

**Etymology.** The specific epithet is the Latin adjective *genuīna* (meaning, “genuine”).

**Diagnosis.** As for the genus (vide supra).

**Materials.** Holotype, a 2nd or 3rd instar male, No. CNU-PHA-MA2016019; Paratype, a 2nd or 3rd instar female, No. CNU-PHA-MA2016020.

**Locality and horizon.** All the amber specimens described herein were legally acquired by Mr Fangyuan Xia before 2015 and donated for this study in 2016 (before June 2017). All the amber specimens were collected from Kachin (Hukawng Valley) of northern Myanmar, which was dated at 98.79 ± 0.62 Ma (mid-Cretaceous, Cenomanian) [26, 27].

**Remarks. ***Breviala* gen. nov. and *Electroclavella* gen. nov. differ from extant *Timema* by the antenna longer than body, scape not stouter, abdomen not surrounded by extensive membrane, the mesal lobe on the right cercus of male absent, and cercus simple and strongly elongate [28, 29]; differ from *Electrotimema* (Baltic amber) by the compound eye large, leg slender and tibia without spines ventroapically, the mesal lobe on the right cercus of male absent, and cercus straight and elongate [30]; differ from *Tumefactipes* and *Granosicorpes* (Kachin amber) by the prothorax short, legs slender without spines ventrally and ventroapically, epiproct triangular, and arolia present [31]. In addition, *Breviala* differs from *Electroclavella* mainly by the head longer than wide, prothorax short, wing buds present, thoracic terga surrounded by extensive membrane, hind legs shorter than abdomen, and epiproct large.

(See Additional file 1 for complete descriptions of *Breviala cretacea* gen. et sp. nov. and *Electroclavella genuina* gen. et sp. nov.)

## Discussion

### Phylogenetic positions of *Breviala* and *Electroclavella*

**Fig. 4:**
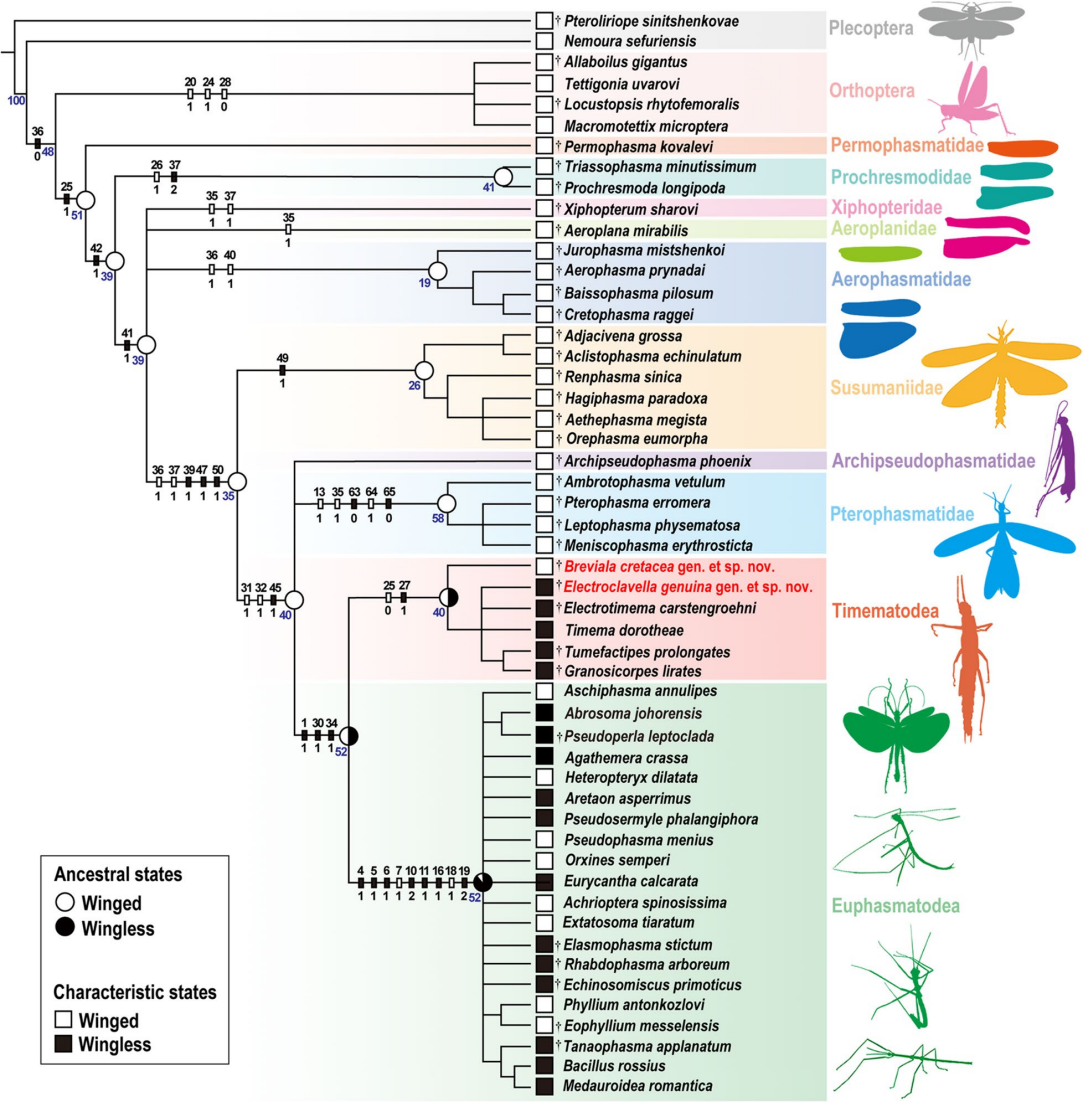
Phylogeny of Holophasmatodea, showing the placements of *Breviala cretacea* and *Electroclavella genuina*, and the wing characteristic and ancestral states, see Additional file 1: Figs. S2 and S3 for details of the strict consensus tree and ancestral character state reconstruction of wings. The numbers under the branch nodes are bootstrap support values (data in blue). “†” indicate the fossil species

The newly discovered and described genera *Breviala* and *Electroclavella* from mid-Cretaceous Kachin amber share five synapomorphies of Phasmatodea: labrum emarginate, prothoracic defensive glands present, metasternum and abdominal sternum I fused, area apicalis of tibiae present, and cerci undivided [22, 28]. They can be further assigned to Timematodea based on the combination of the body not elongate, the median line present on the thorax and abdomen, abdominal tergum I not fused with the metanotum, tarsus pseudotrimeric, and ungues asymmetrical [29]. At present, most genera of Timematodea, including *Timema*, *Electrotimema*, *Tumefactipes*, *Granosicorpes*, and *Electroclavella* are wingless. *Breviala cretacea* gen. et sp. nov. is the only species that retains wings, and based on the analysis presented here, is recovered as the earliest-diverging lineage of Timematodea (Fig. 4, Additional file 1: Fig. S2), a position critical for the reconstruction of patterns of wing evolution in early Phasmatodea.

### Phylogenetic analysis of Holophasmatodea

**Fig. 5:**
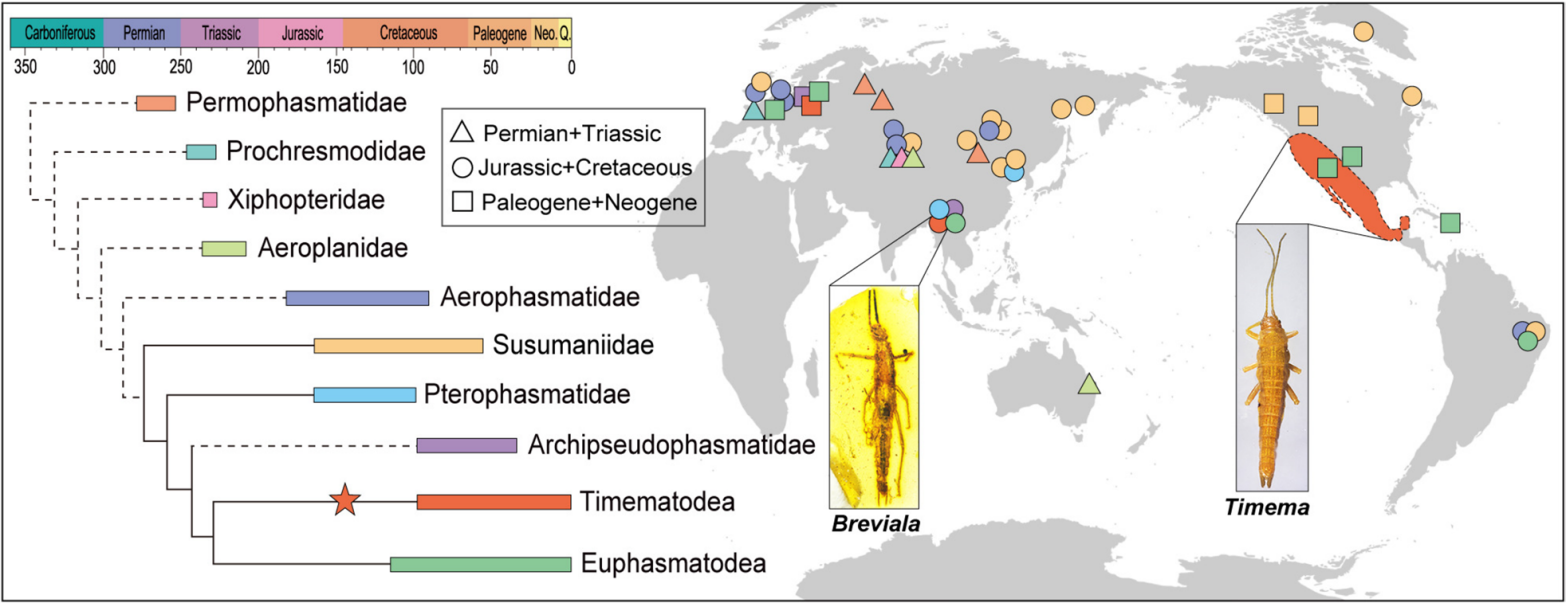
Hypothetical evolutionary relationships within Holophasmatodea, with clade occurrences mapped across biogeographical regions and geographical epochs, source data are listed in Additional file 1: Table S3. Colors represent the distribution of families and triangles, circles, and squares represent different geological ages. The red area on the right represents the geographical distribution of extant *Timema*. The star represents the clade of Timematodea

We carried out a phylogenetic analysis by using morphological characters from representatives of all major living and fossil lineages of Holophasmatodea, with the goal of confirming the taxonomic position of the new taxa, elucidating relationships among all families of Phasmatodea, and from which to explore patterns of wing evolution. In the strict consensus tree (Fig. 4, Additional file 1: Fig. S2), most fossil groups of Holophasmatodea form a grade to Phasmatodea s.l., while Phasmatodea s.l. was recovered as monophyletic, as were some of the individual families within the basal grade. Most critically, Susumaniidae, Pterophasmatidae, Timematodea, and Euphasmatodea were all recovered as monophyletic, including the latter two as sisters. However, the internal relationships of Euphasmatodea could not be recovered based on the phylogenetic analysis. The phylogeny also provided rather interesting insights into the progressive relationships of extinct Holophasmatodea (Fig. 5), particularly the potential relative placements for Permophasmatidae, Prochresmodidae, Xiphopteridae, Aeroplanidae, Aerophasmatidae, and Archipseudophasmatidae, although further study is needed as much of this fossil material is highly fragmentary and more data are needed from specimens with a greater suite of body characters [16]. Archipseudophasmatidae are no longer considered a monophyletic group based on our phylogenetic analysis, and their former constituents were found to belong to lineages having fully-developed wings (Susumaniidae/Pterophasmatidae) or were nested within Euphasmatodea (*Pseudoperla leptoclada* of Archipseudophasmatidae was recovered within Euphasmatodea). Archipseudophasmatidae require much further study. The results suggest that Permophasmatidae, Prochresmodidae, Xiphopteridae, Aeroplanidae, and Aerophasmatidae are stem groups of Phasmatodea (as the larger group Holophasmatodea), and the definitive Phasmatodea s.l. comprise the extinct Susumaniidae and Pterophasmatidae, along with the extant sister clades Timematodea and Euphasmatodea (Fig. 4).

### Palaeogeographic distribution of Holophasmatodea

The earliest putative stem-group stick insects of the Permophasmatidae are largely reported from the Permian strata of Mongolia and Russia [32, 33], suggesting that Holophasmatodea might have originated in Eurasia during the Permian Period. The younger stem-group families Prochresmodidae, Xiphopteridae, and Aeroplanidae are known from the Late Triassic of Kyrgyzstan, France, and Australia [8, 32, 34, 35], revealing that these groups were already widespread at the time of Pangean emergence [36]. During the Jurassic and Cretaceous Aerophasmatidae, Susumaniidae, and Pterophasmatidae were similarly widespread and flourished until the Eocene [16, 17, 24, 37–39]. Fossils of Timematodea are known from mid-Cretaceous Kachin amber and Eocene Baltic amber [30, 31], showing a formerly broad distribution relative to modern *Timema*. The earliest fossil record for Euphasmatodea is from the Early Cretaceous of Brazil [40], while there is also a considerable abundance of species from mid-Cretaceous Kachin amber [17, 21, 41], and others are also known from the Cenozoic of North America and Germany, and in Baltic and Dominican amber [42–44]. The temporal and biogeographic occurrence of the extinct lineages (Fig. 5) demonstrates that phasmatodeans have a long evolutionary history, perhaps as far back as the Late Permian. Most extant species are distributed in tropical and subtropical regions [18], and this accords with the high occurrence of stick insects in Kachin amber, which is well known to have been a humid tropical environment during the Cretaceous [36, 45].

Extant Timematodea (*Timema*) are distributed in western North America (California and adjacent regions) [46], while amber fossils of Timematodea have been discovered in the mid-Cretaceous of northern Myanmar as well as in mid-Eocene Baltic [30, 31]. These amber fossils reveal a formerly widespread distribution for the lineage, with substantial extinction in diversity and particularly geographic coverage, leaving a today relict *Timema* (Fig. 5). The earliest known fossil occurrences for Timematodea are currently those from mid-Cretaceous Kachin amber. All other fossils of Phasmatodea from this same deposit are of the Pterophasmatidae and Euphasmatodea, while Susumaniidae have not yet been documented. However, abundant impression fossil species of Susumaniidae, but no Timematodea or Euphasmatodea, have been reported in the Mesozoic of Asia, reflecting a clear faunal difference between northern Myanmar and northeastern China in the Middle Jurassic to mid-Cretaceous [47, 48]. During the mid-Cretaceous, the West Burma Block comprised a somewhat isolated island surrounded by sea, possessed a warm climate, and was not connected to the mainland at the time [36, 49–51]. It is inferred that Timematodea were most likely widespread prior to this, dispersing across Eurasia and into what would eventually become Europe as well as into western North America before the West Burma Block separated from Gondwana in the Late Jurassic [36, 50]. Accordingly, Timematodea almost assuredly have a long evolutionary history that extends at least into the Late Jurassic, even though representatives of Timematodea have yet to be discovered in Jurassic deposits. Given that many of their defining characters are minute and difficult to observe in anything but the finest-preserved compressions, distinguished Jurassic Timematodea from other early Phasmatodea may prove challenging.

### Wing evolution of Holophasmatodea

Timematodea have traditionally been considered to be an exclusively wingless lineage, an assumption that would appear safe based on a superficial interpretation of extant species alone. However, both male and female nymphs of *Breviala cretacea* with wing buds demonstrate that some species of Timematodea once had wings, at least during the mid-Cretaceous. The wing buds of *B. cretacea* were obviously extensions of the meso- and metanota (Figs. 1J–M and 2F, G), and were completely lost in other Timematodea. The wingless species of *Electroclavella*, *Tumefactipes*, and *Granosicorpes* from the same deposit demonstrate that wings among Timematodea had already begun regression by the mid-Cretaceous. Based on the phylogenetic analysis, the ancestral condition for Timematodea was most likely fully developed wings with strong flight capability, given that the next more ancestral node had such wings plesiomorphically. This would accord with the former distribution of Timematodea, which would have necessitated considerable flight ability. Subsequently, the wings of Timematodea became reduced and ultimately lost, leading to a relict distribution for extant *Timema*.

The more ancient lineages of Holophasmatodea, including Permophasmatidae, Prochresmodidae, Xiphopteridae, Aeroplanidae, Aerophasmatidae, Susumaniidae, and Pterophasmatidae, all had fully developed membranous fore- and hind wings, and based on their construction, were likely strong flyers, with forewings longer than or equal to the hind wings (Fig. 4). Timematodea and Euphasmatodea by contrast possessed vestigial wings or lack them entirely. Wings in these clades are mainly expressed as (i) the moderately sclerotized forewing (tegmina), shorter than the hind wing remigium with a membranous anal field (with weak flight ability); (ii) almost entirely sclerotized and tiny forewing and hind wing without anal field (without flight capability); (iii) forewing absent and sclerotized hind wing remigium with a membranous anal field (with weak flight ability); (iv) wingless (both fore- and hind wing absent). Interestingly, Phasmatodea is the only insect group that has the forewing absent but the hind wing present with weak flight capability, for example, *Aschiphasma* (Aschiphasmatodea), the sister group to Neophasmatodea [20, 25]. In conclusion, the common ancestors of Holophasmatodea were fully winged, and the sclerotization, reduction, and loss of the forewings might be earlier than that of the hind wing during the evolution of Phasmatodea. A similar pattern can also be observed in Orthoptera. Ancestors of Orthoptera were all fully winged, and most fossil species of Orthoptera from the Mesozoic had membranous forewings longer than or equal to the hind wings [47], but extant orthopterans have moderately sclerotized forewings shorter than the hind wings or can be wingless.

Similarly, the phylogenetic analysis with the ancestral-state reconstruction reveals that the common ancestors of Holophasmatodea were winged, which is consistent with all fossil evidence. While the ancestral states of Timematodea + Euphasmatodea were probably half-winged, the brachypterous and wingless lineages (Timematodea and Euphasmatodea) obviously appeared later. The Polyneoptera are a monophyletic group supported by abundant morphological and molecular evidence [1, 23], and it is generally assumed that wings arose once [3]. While wings have evolved a single time among insects, they became vestigial or lost numerous times. The ancestors of most groups of Polyneoptera had fully developed wings, even stem-group Notoptera were winged relative to their extant suborders (Grylloblattodea and Mantophasmatodea), which likely lost wings once in their common ancestor [52, 53]. Uniquely, the ancestors of Holophasmatodea had fully developed wings, however, independent reductions and losses of wings occurred in the extant lineages Timematodea and Euphasmatodea. Given this, it is best to interpret wing evolution in Phasmatodea simply as the regression of wings.

Most extant stick and leaf insects feed on and mimic angiosperms, but the host ranges of extant *Timema* include abundant conifers such as cypresses and pines [25]. The mouthparts of *B. cretacea* and *E. genuina* were well-preserved and similar to extant Timema (Figs. 1F and 3F, H), indicating that their feeding habits were likely similar and conserved despite the considerable time separating them, although it must be noted that their mouthparts are quite generalized chewing mouthparts common to many Polyneoptera and do not indicate specializations for any given floral host. Before the establishment of angiosperm-dominated forests, the morphologies of Mesozoic *Aclistophasma echinulatum* and *Cretophasmomima melanogramma* revealed that some early Phasmatodea had specialized on ferns and gymnosperms [16, 54]. Angiosperms rose to prominence during the Cretaceous, providing a new floristic environment and diversity of resources for stick insects, aiding the co-diversification of these new lineages [55]. Based on all available fossil evidence, the Cretaceous appears to have been a key period for the reduction and loss of wings in Phasmatodea, as well as a shift between older and younger lineages within the clade (Fig. 5). Those fully winged stem groups began to disappear and be replaced during the Cretaceous this occurred alongside the floristic shift. Such a faunal transition seems to have come about with a shift in anti-predation strategy away from flight capability and toward more elaborate mimicry. The different forms of vestigial wings are used for purposes other than flying, mainly defensive and mimetic functions in extant clades. It is therefore clear that camouflage proved a more effective means of defense in Phasmatodea, relative to flight. Such a strategy was apparently achieved numerous times as these non-aggressive, phytophagous, and sometimes fragile insects specialized on a growingly flowered world since the Cretaceous, gradually abandoning the gossamer wings that had otherwise carried insects to success since the Devonian.

## Conclusions

We report new mid-Cretaceous fossils of Timematodea, supporting independent wing reductions and losses among Timematodea and Euphasmatodea. Through the ancestral-state reconstruction of Holophasmatodea, our results demonstrate that common ancestors of Phasmatodea were winged, and the brachypterous and wingless lineages appeared later. The pattern of wing evolution can be simply interpreted as the regression of wings, probably since the Cretaceous, which could be closely related to a shift in anti-predation strategies.

## Methods

### Material preservation and imaging

The specimens studied are deposited in the Key Lab of Insect Evolution and Environmental Changes, College of Life Sciences, Capital Normal University, Beijing, China (CNUB; Dong Ren, Curator). All specimens were examined and photographed by using a Nikon SMZ 25 microscope with an attached Nikon DS-Ri2 digital camera system as well as a Nikon ECLIPSE Ni microscope with an attached NikonDS-Ri2 digital camera system. The green background photographs were taken with the Zeiss LSM 780 inverted confocal laser microscope (CLM) equipped with 488 nm laser.

### Phylogenetic analysis

For the phylogenetic analysis, we chose two species of Plecoptera and four species of Orthoptera as outgroups, and 46 species of Holophasmatodea (= Phasmatodea + extinct stem groups) as ingroups, inclusive of fossil representing all extinct clades and most extant clades. A total of 71 body characters are listed in Additional file 1: Table S1, and the character-state matrix consisting of 52 taxa and 71 characters is provided in Additional file 1: Table S2. The result of the phylogenetic analysis produced 12 most parsimonious trees, from which strict consensus was produced (Fig. 4 and Additional file 1: Fig. S2).

Parsimony analysis was performed using WinClada (Version 1.00.08) and NONA (Version 2.0) [56, 57]. Tree search implemented a heuristic search method and the options were set to hold 10,000 trees, 1000 replications, 100 starting tree replications, and a multiple TBR + TBR search strategy. All characteristics were considered unordered and weighted equally. Bootstrap supporting values, determined by using NONA with 1000 replications, are represented as numbers under the branch nodes (Fig. 4). Ancestral character state reconstruction of wings (Additional file 1: Fig. S3) was conducted using equally weighted parsimony methods in Mesquite 3.81 [58].

### Supplementary Information

**Additional file1: Dataset S1.** Systematic palaeontology. **Figure S1.** Paratype of *Electroclavella genuina*. **Figure S2.** The strict consensus tree of phylogenetic analysis. **Figure S3.** Ancestral character state reconstruction of wings. **Table S1.** Definition of characters and their states. **Table S2.** Character state matrix of 71 characters for the 52 taxa included in the phylogenetic study. **Table S3.** The list of described Phasmatodea fossils.

**Additional file 2:** Character state matrix of phylogenetic study.

## Data Availability

All data generated or analyzed during this study are included in this published article and its supplementary information files. Nomenclatural acts established herein are registered in ZooBank (www.zoobank.org) following the requirements of the International Code of Zoological Nomenclature and listed under LSID: urn:lsid:zoobank.org:pub:8E0870FD-6D9F-4DB6-BEB9-49637E52516F.

## Abbreviations

Af: Antennal field
Apg: Aperture of pronotal gland
Ar: Arolium
Ce: Cercus
Cly: Clypeus
Ep: Epiproct
Eu: Euplantula
Fc: Frontal convexity
Fla1: Flagellomere I
FW: Forewing
Ga: Galea
Gap8: Gonapophyses VIII
Ge: Gena
Gl: Glossa
HW: Hind wing
Inc: Incisor
Lat: Lacinia teeth
Lb: Labrum
Me: Mentum
Md: Mandible
Mst: Mesothorax
Mtt: Metathorax
Pa: Paraproct
Pd: Pedicellus
Pgl: Paraglossa
Plb: Labial palpus
Pme: Prementum
Pmx: Maxillary palpus
Prt: Prothorax
Sc: Scape
S1, S8, and S9: Abdominal sterna I, VIII, and IX
Sme: Submentum
T1, T9 and T10: Abdominal terga I, IX and X
Ta1–Ta3: Tarsomeres I–III
Un: Pretarsal claw (ungues)
