# Supplementary material for: Independent wing reductions and losses among stick and leaf insects (Phasmatodea), supported by new Cretaceous fossils in amber
Source: BMC Biol. 2023 Oct 9;21:210. doi: 10.1186/s12915-023-01720-0 (PMC10561512; doi:10.1186/s12915-023-01720-0)
Supplement: Supplementary file 1 — Additional file1: Dataset S1. Systematic palaeontology. Figure S1. Paratype of Electroclavella genuina. Figure S2. The strict consensus tree of phylogenetic analysis. Figure S3. Ancestral character state reconstruction of wings. Table S1. Definition of characters and their states. Table S2. Character state matrix of 71 characters for the 52 taxa included in the phylogenetic study. Table S3. The list of described Phasmatodea fossils. [file 12915_2023_1720_MOESM1_ESM.docx]

**Additional file 1**

**Independent wing reductions and losses among stick and leaf insects (Phasmatodea), supported by new Cretaceous fossils in amber**

Hongru Yang^1^, Michael S. Engel^2^, Chungkun Shih^1,3^, Fan Song^4^, Yisheng Zhao^4^, Dong Ren^1^, Taiping Gao^1*^

^1^ College of Life Sciences, Capital Normal University, Beijing 100048, China.

^2^ Division of Invertebrate Zoology, American Museum of Natural History, New York, NY 10024, USA.

^3^ Department of Paleobiology, National Museum of Natural History, Smithsonian Institution, Washington, DC 20013-7012, USA.

^4^ Department of Entomology MOA Key Lab of Pest Monitoring and Green Management, College of Plant Protection, China Agricultural University, Beijing 100193, China

*Corresponding author. Email: tpgao@cnu.edu.cn

**Contents**

Dataset S1. Systematic palaeontology

Figure S1. Paratype of *Electroclavella genuine* Yang, Engel, Shih & Gao gen. et sp. nov.

Figure S2. The strict consensus tree of phylogenetic analysis.

Figure S3. Ancestral character state reconstruction of wings.

Table S1. Definition of characters and their states.

Table S2. Character state matrix of 71 characters for the 52 taxa included in the phylogenetic study.

Table S3. The list of described Phasmatodea fossils.

**Dataset S1. Systematic palaeontology**

Order Phasmatodea Jacobson & Bianchi, 1902

Suborder Timematodea Kevan, 1977

Family Timematidae Caudell, 1903

***Breviala* Yang, Engel, Shih & Gao gen. nov. (Figs. 1 and 2)**

***Breviala cretacea* Yang, Engel, Shih & Gao sp. nov.**

**Holotype.** No. CNU-PHA-MA2016017.

**Description.** Body robust, surface smooth, wing buds present. Head ovoid, longer than width, prognathous, dorsoventrally flattened; anterolateral regions of the frons somewhat swollen; clypeus large, protruding; labrum weakly emarginate distally; mandible wedge-shaped, with incurved lateral margins, incisor and molar lobes visible; maxilla with two lacinial teeth; galea broad, round, overlapping the lacinia; compound eye ovoid, exophthalmic, about 1/3 as long as head; ocelli absent; lateral regions of the gena swollen; antenna filiform, with eight antennomeres (as preserved); antennifer well-developed; scape cylindrical, slender; pedicel broad, about 1/3 as long as scape; all flagellomeres slightly longer and narrower than pedicel (as preserved); gula lacking; cervix membrane and cervical sclerites present, the first cervical sclerite elongated and narrow; the second and dorsal cervical sclerite unclear.

Thoracic segments lacking elongation, median line present; thoracic terga simple, without lateral extension, surrounded by extensive membrane between pro- meso- and metanotum; pronotum rectangular, similar length with head and mesonotum; prothoracic defensive glands present; wing buds of meso- and metathorax obviously; mesonotum square, longer than metanotum; metanotum wider than length; abdominal tergum I (‘median segment’) not associated with metanotum; thoracic sterna mostly membranous; metasternum associated with abdominal sternum I.

Legs slender and shorter than abdomen; trochanter fused with femur; all femora and tibiae round in cross section; profemora straight at the base; fore and middle legs almost equal in length, about 2/3× as long as hind legs; V-shaped area apicalis of tibiae present; tarsus pseudotrimeric, tarsomere I similar to tarsomere III in length; tarsomere II shorter than I and III; basitarsomere with three attachment pads (euplantulae), the third euplantulae expanded; euplantulae of tarsomere II strongly expanded and separated into two parts; pretarsal unguis and arolia present, asymmetrical; with the anterior unguis slightly smaller than the posterior one.

Abdomen not elongate, narrower than thorax, eleven abdominal segments observed and median line present; segments I–IV of subequal size, remaining segments gradually narrowing, all segments with similar length; segments V–VII of subequal size; segments VIII–X of subequal size and narrower than segments V–VII; segment X posterior margin projecting, slightly raised; epiproct large, lamellar, triangular, exceeding the posterior margin of segment X; paraproct symmetrical and somewhat longer than epiproct; cercus unsegmented, strongly elongate, cylindrical, gradually tapering toward apex.

**Measurements (****length** **in mm).** Body about 18.17 (excluding antennae); head 1.92; left antenna 4.12, right antenna 3.86 (as preserved); scape 0.92; pedicel 0.37; flagellomere I 0.58; prothorax 1.86; mesothorax 1.81; metathorax 1.51; abdomen 10.26; cercus 1.59; profemur 2.04; protibia 1.52; protarsus 1.40; mesofemur 2.13; mesotibia 1.69; mesotarsus 1.41; metafemur 3.69; metatibia 3.45; metatarsus 1.76.

**Paratype.** No. CNU-PHA-MA2016018.

**Description.** Body features of female similar with those of male. Head poorly preserved, slightly longer than width; labrum, compound eye visible, antenna longer than body; maxillary palpus pentamerous; labial palpus trimerous. Wing buds of meso- and metathorax present; leg slightly slender than that of male. Operculum, gonapophyses visible; cercus poorly preserved.

**Measurements (length in mm).** Body about 13.82 (excluding antennae); head 1.82; left antenna 11.63, right antenna 11.90 (as preserved); scape 0.68; pedicel 0.31; flagellomere I 0.43; prothorax 1.37; mesothorax 1.19; metathorax 0.96; abdomen 7.64; cercus 0.69 (as preserved); profemur 2.49; protibia 1.61; protarsus 1.13; mesofemur 2.13; mesotibia 1.55; mesotarsus 1.24; metafemur 3.43; metatibia 2.97; metatarsus 1.38.

***Electroclavella* Yang, Engel, Shih & Gao gen. nov. (Fig. 3 and fig. S1)**

***Electroclavella* *genuina* Yang, Engel, Shih & Gao sp. nov.**

**Holotype.** No. CNU-PHA-MA2016019.

**Description.** Body robust, wingless. Head globular, prognathous, anterolateral regions of the frons and posterolateral genae swollen, slightly raised posteriorly; clypeus large, trapezoidal; labrum weakly emarginate distally, shorter and narrower than clypeus; maxillary palpus pentamerous; labium obviously, glossa and paraglossa long and narrow, almost equal length apically, labial palpus trimerous; prementum small, with a shallow median sulcus, bifurcates proximally; mentum with two triangular sclerites; submentum sclerotized, cuspidal apically; compound eye nephroid, slightly exophthalmic; ocellus absent; antenna filiform, with 32 antennomeres (left antenna), longer than body; antennifer visible; scape cylindrical; pedicel about 1/2 as long as scape; the first flagellomere longer than others, about 4× as long as pedicel; gula lacking; cervix membrane; cervical sclerites unclear.

Thoracic segment not elongate, median line visible; thoracic terga surrounded by extensive membrane; pronotum rectangular, equal to the head in length and longer than meso- and metanotum; prothoracic defensive glands obviously; wing buds of meso- and metathorax absent; mesonotum square, similar length with metanotum; metanotum slightly wider than mesonotum; abdominal tergum I (‘median segment’) visible; metasternum fused with abdominal sternum I.

Leg slender and longer than abdomen; trochanter fused with femur; profemora straight at the base; the length of fore and middle legs almost equal; hind legs longer and about 2× as long as middle legs; all femora and tibiae round in cross section; the length of femur and tibia similar in each leg; V-shaped area apicalis of tibiae present; tarsus pseudotrimeric, tarsomere I similar to tarsomere III in length; tarsomere II shortest; basitarsomere with three euplantulae, the euplantulae I and II small and narrow; the third euplantulae expanded and divided longitudinally; euplantulae of tarsomere II strongly expanded and separated two parts; euplantulae of tarsomere III very small and not obviously; pectinate ungues visible, the anterior unguis shorter than the posterior one; arolia large and longer than ungues.

Abdomen slightly narrower than thorax, eleven abdominal segments observed and median line present; segments I, II of subequal width and I shorter than II, remaining segments gradually narrowing; segments III–VII of subequal size, V slightly longer than others; segments VIII, IX of subequal length, and VIII wider than IV; segment X longer but narrower than others; epiproct small, triangular, exceeding the posterior margin of segment X; paraproct symmetrical and slightly longer than epiproct; cercus unsegmented, strongly elongate, inconsistent width, gradually tapering toward apex; the sternum IX of male a slight bulge.

**Measurements (length in mm).** Body about 11.98 (excluding antennae); head 1.63; left antenna 11.45, right antenna 6.16 (as preserved); scape 0.48; pedicel 0.28; flagellomere I 1.21; prothorax 1.44; mesothorax 0.99; metathorax 1.03; abdomen 6.51; cercus 1.09; profemur 2.26; protibia 1.55; protarsus 1.16; mesofemur 1.88; mesotibia 1.33; mesotarsus 0.95; metafemur 3.32; metatibia 2.85; metatarsus 1.23.

**Paratype.** No. CNU-PHA-MA2016020.

**Description.** Body features of female similar with those of male, slightly narrower than male. Head, thorax, legs and abdomen well-preserved; labrum, mandibles, maxillae, labium unclear; maxillary palpus pentamerous obviously. Wing buds of meso- and metathorax absent; area apicalis of tibiae visible; pseudotrimeric tarsus, pectinate ungues, euplantulae and arolia obviously. Female with operculum, gonapophyses IIX, IX (valve I, II), gonoplac (valve III) visible; epiproct, paraproct, cercus well-preserved.

**Measurements (length in mm).** Body about 13.31 (excluding antennae); head 1.48; left antenna 14.13, right antenna 3.61 (as preserved); scape 0.69; pedicel 0.30; flagellomere I 1.73; prothorax 1.54; mesothorax 1.20; metathorax 1.06; abdomen 7.93; cercus 1.40; profemur 2.38; protibia 1.87; protarsus 1.28; mesofemur 2.21; mesotibia 1.89; mesotarsus 1.20; metafemur 3.90; metatibia 3.47; metatarsus 1.73.


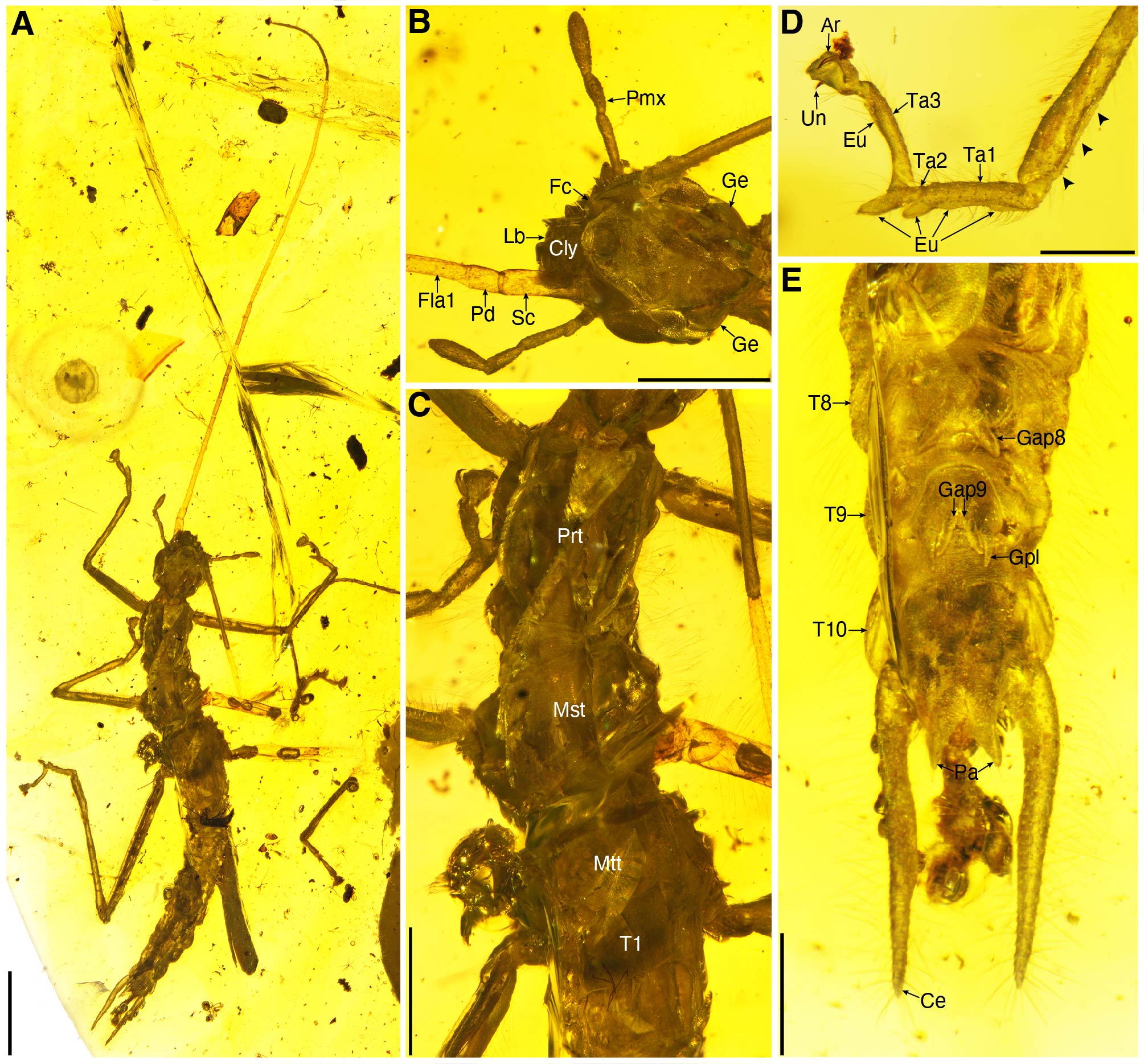


**Figure S1.** Paratype of *Electroclavella genuina*, No. CNU-PHA-MA2016020. **A** Habitus in dorsal view. **B** Head in dorsal view. **C** Thorax in dorsal view. **D** Metatarsus in lateral view. **E** Female genitalia in ventral view. Scale bars: **A** 2 mm; **B**, **C** 1 mm; **D**, **E** 0.5 mm.


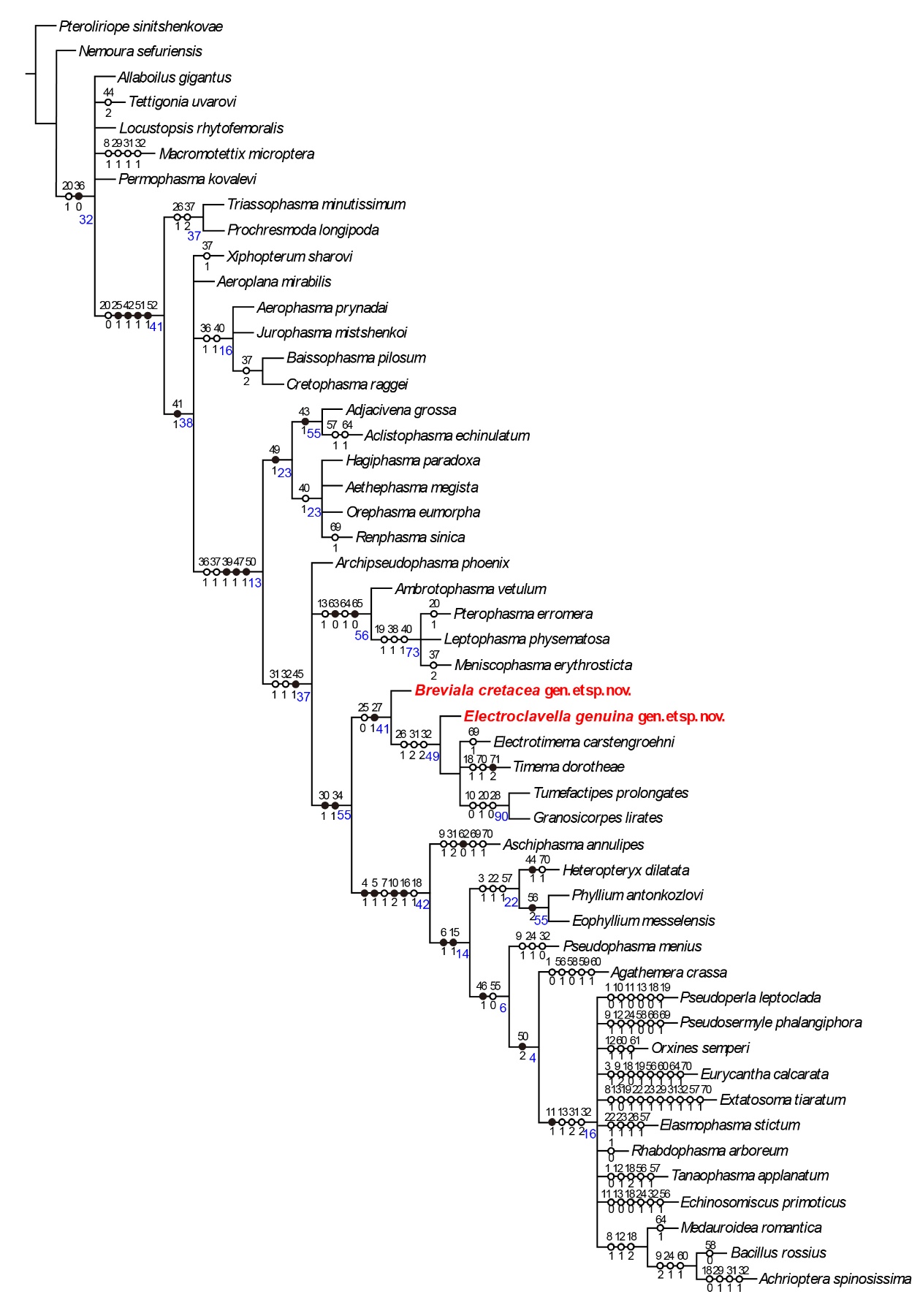


**Figure S2.** The strict consensus tree of phylogenetic analysis. Tree length = 192 steps, consistency index (CI) = 0.43, retention index (RI) = 0.77.


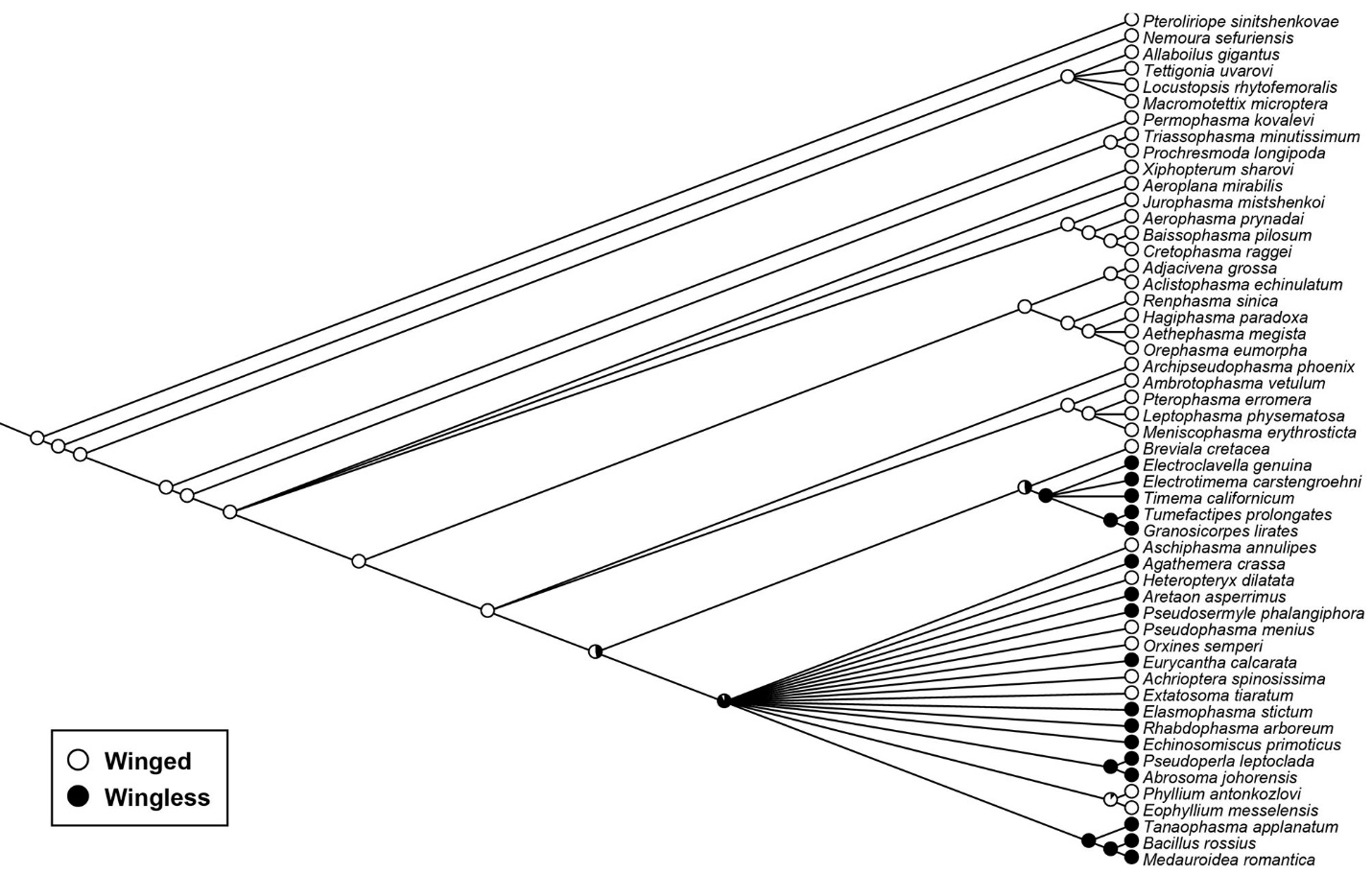


**Figure S3.** Ancestral character state reconstruction of wings.

**Table S1.** Definition of characters and their states.

No. Characters and their states

| 1. Compound eyes size: 0->1/3 of head; 1-<1/3 of head. 2. Labrum: 0-not emarginated; 1-emarginated. 3. Incisor lobe of mandible: 0-crenate; 1-entire. 4. Molar lobe of mandible: 0-toothed lobe; 1-cusp. 5. Apical lacinial teeth: 0-two; 1-three. 6. Galealobulous: 0-absent; 1-present. |
| --- |
| 1. Antennifer: 0-well-defined; 1-vestigial. |
| 1. Antenna: 0-longer than profemora; 1-shorter than profemora. |
| 1. Cervix: 0-membranous; 1-with two lateral plates or a median plate; 2-gula. |
| 1. Prothorax: 0-longer than meso- and metathorax; 1-similar length with meso- and metathorax; 2-shorter than meso- and metathorax. 2. Mesothorax: 0-not elongated; 1-elongated. 3. Metathorax: 0-not elongated; 1-elongated. 4. Length/width of thorax: 0-<4; 1->4. |
| 1. Defensive glands of prothorax: 0-absent; 1-present. 2. Position of defensive glands: 0-on the front edge of the pronotum; 1- below the front corners of the pronotum. |
| 1. Metanotum and abdominal tergum I: 0-separated; 1-fused. |
| 1. Metasternum and abdominal sternum I: 0-separated; 1-fused. |
| 1. Legs: 0-fore and middle legs shorter than hind legs; 1-similar length; 2-forelegs longer than middle and hind legs. |
| 1. The base of profemora: 0-straight; 1-slightly curved; 2-strongly curved. |
| 1. Metafemora: 0-not or only slightly stronger than the other femora; 1-conspicuously thickened and reinforced. |
| 1. Trochanter: 0-movably detached from the femur; 1-fused with the femur. |
| 1. Lateral extension of thorax: 0-absent; 1-present. |
| 1. Lateral extension of legs: 0-absent; 1-present. |
| 1. Area apicalis of tibiae: 0-present; 1-absent. |
| 1. Tarsus: 0-3 or 4 articles; 1-5 articles. |
| 1. Basitarsus: 0-shorter than the next tarsomeres together; 1-as long as or longer than the next tarsomeres together. 2. Ungues: 0-symmetrical; 1-asymmetrical. 3. Arolium: 0-absent; 1-present. 4. Wing: 0-winged; 1-wingless. 5. The length of wings: 0-forewing longer than or equal to hind wing; 1-forewing shorter than hind wing or absent. |
| 1. Forewing: 0-well-developed, reaching the end of abdomen; 1-shorter, not reaching the end of abdomen; 2-absent. 2. Hind wings: 0-well-developed, reaching the end of abdomen; 1-shorter, not reaching the end of abdomen; 2-absent. |
| 1. Cross-veins of wings: 0-abundant, forming reticulate venation; 1-few. |
| 1. Position of forewing in mesothorax: 0-in the basal half of mesothorax; 1-in the distal half of mesothorax. 2. Length/width of forewing: 0-<5; 1->5. 3. Precostal area of forewing and hindwing: 0-present; 1-absent. |
| 1. RP of forewing: 0-more than three branches; 1-two or three branches; 2-unbranched. |
| 1. Branch location of RP: 0-in proximal of RP origin; 1-in distal of RP origin. |
| 1. M of forewing: 0-three or more than three branches; 1-two branches; 2-unbranched or without M. |
| 1. Branch location of M: 0-basal to RP origin; 1-distal to RP origin. 2. Branches of MA in forewing: 0-branched; 1-simple. 3. Branches of MP in forewing: 0-branched; 1-simple. 4. MP of forewing: 0-not approaching CuA; 1-approaching CuA. |
| 1. Cu of forewing: 0-more than three branches; 1-three branches; 2-less than three branches. |
| 1. Anal area of forewing: 0-wide; 1-narrow. |
| 1. R of hind wing: 0-branched; 1-unbranched or without R. |
| 1. RP of hind wings: 0-branched; 1-unbranched. |
| 1. Branch location of R: 0-in proximal of hind wings; 1-in distal of hind wings. 2. RP+MA in hind wing: 0-not fused; 1-fused. |
| 1. M of hind wings: 0-three or more than three branches; 1-two branches; 2-unbranched or without M. 2. Branches of MA in hind wing: 0-branched; 1-simple. 3. Branches of MP in hind wing: 0-branched; 1-simple. |
| 1. Cu of hind wing: 0-more than three branches; 1-two branches; 2-unbranched or without Cu. |
| 1. CuA+CuP fused apically on hind wing: 0-absent; 1-present. |
| 1. 2–7A of hind wing: 0-without a common origin at the wing base; 1-with a common origin at the wing base. 2. Abdomen: 0-uniform; 1-compressed, wider in anterior and narrowed toward to posterior; 2-leaf-shape. 3. Lateral extension of abdomen: 0-absent; 1-present. |
| 1. Sternum VIII (operculum) of females: 0-short, not covering the ovipositor valves; 1-long, at least beyond the ovipositor valves. |
| 1. Gonapophyses VIII and IX: 0-present; 1-reduced. |
| 1. Gonoplac: 0-present; 1-reduced. |
| 1. Length of ovipositor: 0-short and reduced, not protruding the abdomen end; 1-gonapophysis VIII or all ovipositor strongly elongated. |
| 1. Subgenital plate (abdominal sternum IX) of the males: 0-undivided; 1-divided transversally. |
| 1. Genitalia of male (phallic organ of abdominal segment IX): 0-with two large phallomeres; 1-phallomeres absent. |
| 1. Abdominal tergum X of males: 0-undivided; 1-split. 2. Abdominal segment X of males: 0-wider than other segments; 1-similar width or narrower than other segments. |
| 1. Thorn pads of tergum X in males: 0-absent; 1-present. |
| 1. Vomer: 0-absent; 1-present. 2. Cercus: 0-segmented; 1-un-segmented. |
| 1. Cercus: 0-straight; 1-curved inward, sickle-shaped. |
| 1. Cercus: 0-circular in cross-section; 1-flatted or flatted at the base, thickened toward the apical. 2. Cercus: 0-single; 1-bipartite; 2-mesal lobe present in right cercus. |

**Table S2.** Character state matrix of 71 characters for the 52 taxa included in the phylogenetic study.

|  | Taxa/ character | 1 | 2 | 3 | 4 | 5 | 6 | 7 | 8 | 9 | 1  0 | 1  1 | 1  2 | 1  3 | 1  4 | 1  5 | 1  6 | 1  7 | 1  8 | 1  9 | 2  0 |
| --- | --- | --- | --- | --- | --- | --- | --- | --- | --- | --- | --- | --- | --- | --- | --- | --- | --- | --- | --- | --- | --- |
| Plecoptera | †*Pteroliriope sinitshenkovae* | ? | ? | ? | ? | ? | ? | ? | 0 | ? | 0 | 0 | 0 | 0 | ? | – | 0 | ? | 0 | 0 | 0 |
|  | *Nemoura sefuriensis* | 0 | 0 | 0 | 0 | ? | 0 | 1 | 0 | 0 | 0 | 0 | 0 | 0 | 0 | – | 0 | 0 | 0 | 0 | 0 |
| Orthoptera | †*Allaboilus gigantus* | ? | ? | ? | ? | ? | ? | ? | ? | ? | 0 | 0 | 0 | 0 | ? | – | ? | ? | 0 | 0 | 1 |
|  | *Tettigonia uvarovi* | 0 | 0 | 0 | 0 | 0 | 0 | 1 | 0 | 0 | 0 | 0 | 0 | 0 | 0 | – | 0 | 0 | 0 | 0 | 1 |
|  | †*Locustopsis rhytofemoralis* | 0 | ? | ? | ? | ? | ? | ? | ? | ? | 0 | 0 | 0 | 0 | ? | – | ? | ? | 0 | 0 | 1 |
|  | *Macromotettix microptera* | 0 | 0 | 0 | 0 | 0 | 0 | 1 | 1 | 0 | 0 | 0 | 0 | 0 | 0 | – | 0 | 0 | 0 | 0 | 1 |
| Permophasmatidae | †*Permophasma kovalevi* | ? | ? | ? | ? | ? | ? | ? | ? | ? | ? | ? | ? | ? | ? | ? | ? | ? | ? | ? | ? |
| Prochresmodidae | †*Triassophasma minutissimum* | ? | ? | ? | ? | ? | ? | ? | ? | ? | ? | ? | ? | ? | ? | ? | ? | ? | ? | 0 | 0 |
|  | †*Prochresmoda longipoda* | ? | ? | ? | ? | ? | ? | ? | ? | ? | ? | ? | ? | ? | ? | ? | ? | ? | ? | 0 | 0 |
| Xiphopteridae | †*Xiphopterum sharovi* | ? | ? | ? | ? | ? | ? | ? | ? | ? | ? | ? | ? | ? | ? | ? | ? | ? | ? | ? | ? |
| Aeroplanidae | †*Aeroplana mirabilis* | ? | ? | ? | ? | ? | ? | ? | ? | ? | ? | ? | ? | ? | ? | ? | ? | ? | ? | ? | ? |
| Aerophasmatidae | †*Aerophasma prynadai* | ? | ? | ? | ? | ? | ? | ? | ? | ? | ? | ? | ? | ? | ? | ? | ? | ? | ? | ? | ? |
|  | †*Jurophasma mistshenkoi* | ? | ? | ? | ? | ? | ? | ? | ? | ? | ? | ? | ? | ? | ? | ? | ? | ? | ? | ? | ? |
|  | †*Baissophasma pilosum* | ? | ? | ? | ? | ? | ? | ? | ? | ? | ? | ? | ? | ? | ? | ? | ? | ? | ? | ? | ? |
|  | †*Cretophasma raggei* | ? | ? | ? | ? | ? | ? | ? | ? | ? | ? | ? | ? | ? | ? | ? | ? | ? | ? | ? | ? |
| Susumaniidae | †*Adjacivena grossa* | 0 | ? | 0 | 0 | ? | ? | 0 | 0 | ? | 1 | 0 | 0 | 0 | ? | ? | 0 | 1 | 0 | 0 | 0 |
|  | †*Aclistophasma echinulatum* | ? | ? | ? | ? | ? | ? | ? | 0 | ? | 1 | 0 | 0 | 0 | ? | ? | 0 | 1 | 0 | 0 | 0 |
|  | †*Hagiphasma paradoxa* | 0 | ? | ? | ? | ? | ? | ? | 0 | ? | 1 | 0 | 0 | 0 | ? | ? | ? | ? | 0 | 0 | 0 |
|  | †*Aethephasma megista* | 0 | ? | ? | ? | ? | ? | ? | ? | ? | 1 | 0 | 0 | 0 | ? | ? | ? | ? | ? | 0 | 0 |
|  | †*Orephasma eumorpha* | ? | ? | ? | ? | ? | ? | ? | ? | ? | 1 | 0 | 0 | 0 | ? | ? | ? | ? | ? | 0 | 0 |
|  | †*Renphasma sinica* | 0 | ? | ? | ? | ? | ? | ? | 0 | ? | 1 | 0 | 0 | 0 | ? | ? | ? | ? | 0 | 0 | 0 |
| Pterophasmatidae | †*Ambrotophasma vetulum* | 0 | ? | 0 | ? | 0 | ? | ? | 0 | ? | 1 | 0 | 0 | 1 | ? | ? | 0 | ? | 0 | 0 | 0 |
|  | †*Pterophasma erromera* | 0 | 1 | ? | ? | ? | ? | 0 | 0 | ? | 1 | 0 | 0 | 1 | ? | ? | ? | 1 | 0 | 1 | 1 |
|  | †*Leptophasma physematosa* | 0 | 1 | ? | ? | ? | ? | 0 | 0 | ? | 1 | 0 | 0 | 1 | ? | ? | ? | 1 | 0 | 1 | 0 |
|  | †*Meniscophasma erythrosticta* | 0 | 1 | ? | ? | ? | ? | 0 | 0 | ? | 1 | 0 | 0 | 1 | ? | ? | ? | 1 | 0 | 1 | 0 |
| Archipseudophasmatidae | †*Archipseudophasma phoenix* | 0 | ? | ? | ? | ? | ? | ? | 0 | ? | 1 | 0 | 0 | 0 | ? | ? | ? | ? | 0 | 0 | 0 |
|  | †*Pseudoperla leptoclada* | 0 | 1 | ? | ? | ? | ? | 1 | 0 | ? | 1 | 0 | 0 | 0 | 1 | ? | 1 | 1 | 0 | 1 | 0 |
| Timematidae | †*Breviala cretacea* | 1 | 1 | 0 | 0 | 0 | 0 | 0 | 0 | 0 | 1 | 0 | 0 | 0 | 1 | 0 | 0 | 1 | 0 | 0 | 0 |
|  | †*Electroclavella* *genuina* | 0 | 1 | ? | ? | ? | ? | 0 | 0 | 0 | 1 | 0 | 0 | 0 | 1 | 0 | 0 | 1 | 0 | 0 | 0 |
|  | †*Tumefactipes prolongates* | 1 | 1 | ? | ? | ? | ? | ? | 0 | ? | 0 | 0 | 0 | 0 | ? | ? | 0 | 1 | 0 | 0 | 1 |
|  | †*Granosicorpes lirates* | 1 | 1 | ? | ? | ? | ? | ? | 0 | ? | 0 | 0 | 0 | 0 | ? | ? | 0 | 1 | 0 | 0 | 1 |
|  | †*Electrotimema carstengroehni* | 1 | ? | ? | ? | ? | ? | ? | 0 | ? | 1 | 0 | 0 | 0 | ? | ? | ? | ? | 0 | 0 | 0 |
|  | *Timema californicum* | 1 | 1 | 0 | 0 | 0 | 0 | 0 | 0 | 0 | 1 | 0 | 0 | 0 | 1 | 0 | 0 | 1 | 1 | 0 | 0 |
| Euphasmatodea | *Aschiphasma annulipes* | 0 | 1 | 0 | 1 | 1 | 0 | 1 | 0 | 1 | 2 | 0 | 0 | 0 | 1 | 0 | 1 | 1 | 1 | 0 | 0 |
|  | *Abrosoma johorensis* | 0 | 1 | 0 | 1 | 1 | 0 | 1 | 0 | 1 | 2 | 1 | 0 | 0 | 1 | 0 | 1 | 1 | 0 | 1 | 0 |
|  | *Agathemera crassa* | 0 | 1 | 0 | 1 | 1 | 1 | 1 | 0 | 0 | 2 | 0 | 0 | 0 | 1 | 1 | 1 | 1 | 1 | 0 | 0 |
|  | *Heteropteryx dilatata* | 1 | 1 | 1 | 1 | 1 | 1 | 1 | 0 | 0 | 2 | 0 | 0 | 0 | 1 | 1 | 1 | 1 | 1 | 0 | 0 |
|  | *Aretaon asperrimus* | 1 | 1 | 1 | 1 | 1 | 1 | 1 | 0 | 0 | 2 | 1 | 0 | 0 | 1 | 1 | 1 | 1 | 1 | 1 | 0 |
|  | *Phyllium antonkozlovi* | 1 | 1 | 1 | 1 | 1 | 1 | 1 | 1 | 0 | 2 | 0 | 0 | 0 | 1 | 1 | 1 | 1 | 1 | 2 | 0 |
|  | *Bacillus rossius* | 1 | 1 | 0 | 1 | 1 | 1 | 1 | 1 | 2 | 2 | 1 | 1 | 1 | 1 | 1 | 1 | 1 | 2 | 2 | 0 |
|  | *Pseudosermyle phalangiphora* | 1 | 1 | 0 | 1 | 1 | 1 | 1 | 0 | 1 | 2 | 1 | 1 | 1 | 1 | 1 | 1 | 1 | 1 | 2 | 0 |
|  | *Pseudophasma menius* | 1 | 1 | 0 | 1 | 1 | 1 | 1 | 0 | 1 | 2 | 0 | 0 | 0 | 1 | 1 | 1 | 1 | 1 | 2 | 0 |
|  | *Orxines semperi* | 1 | 1 | 0 | 1 | 1 | 1 | 1 | 0 | 0 | 2 | 1 | 1 | 1 | 1 | 1 | 1 | 1 | 1 | 2 | 0 |
|  | *Eurycantha calcarata* | 1 | 1 | 1 | 1 | 1 | 1 | 1 | 0 | 2 | 2 | 1 | 0 | 1 | 1 | 1 | 1 | 1 | 0 | 1 | 0 |
|  | *Achrioptera spinosissima* | 1 | 1 | 0 | 1 | 1 | 1 | 1 | 1 | 2 | 2 | 1 | 1 | 1 | 1 | 1 | 1 | 1 | 0 | 2 | 0 |
|  | *Extatosoma tiaratum* | 1 | 1 | 0 | 1 | 1 | 1 | 1 | 1 | 0 | 2 | 1 | 0 | 0 | 1 | 1 | 1 | 1 | 1 | 1 | 0 |
|  | *Medauroidea romantica* | 1 | 1 | 0 | 1 | 1 | 1 | 1 | 1 | 0 | 2 | 1 | 1 | 1 | 1 | 1 | 1 | 1 | 2 | 2 | 0 |
|  | †*Elasmophasma* *stictum* | 1 | 1 | ? | ? | ? | ? | 1 | 0 | ? | 2 | 1 | 0 | 1 | 1 | 1 | 1 | 1 | 1 | 2 | 0 |
|  | †*Rhabdophasma* *arboreum* | 0 | 1 | ? | ? | ? | ? | 1 | 0 | ? | 2 | 1 | 0 | 1 | ? | ? | 1 | ? | 1 | 2 | 0 |
|  | †*Tanaophasma* *applanatum* | 0 | 1 | ? | ? | ? | ? | 1 | 0 | ? | 2 | 1 | 1 | 1 | ? | ? | 1 | ? | 2 | 2 | 0 |
|  | †*Eophyllium messelensis* | ? | ? | ? | ? | ? | ? | ? | ? | ? | ? | 0 | 0 | 0 | ? | ? | ? | ? | 1 | 1 | 0 |
|  | †*Echinosomiscus primoticus* | 1 | ? | ? | ? | ? | ? | ? | 0 | ? | 2 | 0 | 0 | 0 | ? | ? | ? | ? | 0 | ? | 0 |

| **21** | **22** | **23** | **24** | **25** | **26** | **27** | **28** | **29** | **30** | **31** | **32** | **33** | **34** | **35** | **36** | **37** | **38** | **39** | **40** | **41** | **42** | **43** | **44** | **45** | **46** | **47** | **48** | **49** | **50** | **51** | **52** | **53** | **54** | **55** | **56** | **57** | **58** | **59** | **60** | **61** | **62** | **63** | **64** | **65** | **66** | **67** | **68** | **69** | **70** | **71** |
| --- | --- | --- | --- | --- | --- | --- | --- | --- | --- | --- | --- | --- | --- | --- | --- | --- | --- | --- | --- | --- | --- | --- | --- | --- | --- | --- | --- | --- | --- | --- | --- | --- | --- | --- | --- | --- | --- | --- | --- | --- | --- | --- | --- | --- | --- | --- | --- | --- | --- | --- |
| ? | 0 | 0 | ? | 0 | 0 | ? | ? | 0 | ? | 0 | 0 | 0 | 0 | 0 | 1 | 0 | 0 | 0 | 0 | 0 | 0 | 0 | 0 | 0 | 0 | 0 | 0 | 0 | 0 | 0 | 0 | 0 | 0 | 0 | 0 | 0 | – | ? | ? | ? | – | – | ? | 1 | ? | ? | 0 | 0 | 0 | 0 |
| 0 | 0 | 0 | 0 | 0 | 0 | 0 | 1 | 0 | ? | 0 | 0 | 0 | 0 | 0 | 1 | 0 | 0 | 0 | 0 | 0 | 0 | 0 | 0 | 0 | 0 | 0 | 0 | 0 | 0 | 0 | 0 | 0 | 0 | 0 | 0 | 0 | – | 1 | 1 | 0 | – | – | 0 | 1 | 0 | 0 | 0 | 0 | 0 | 0 |
| ? | 0 | 0 | ? | 0 | 0 | ? | ? | 0 | ? | 0 | 0 | 0 | 0 | 0 | 0 | 0 | 0 | 0 | 0 | 0 | 0 | 0 | 0 | 0 | 0 | 0 | 0 | 0 | 0 | 0 | 0 | ? | ? | ? | 0 | 0 | – | ? | ? | 1 | – | – | ? | 1 | ? | ? | ? | ? | ? | ? |
| 0 | 0 | 0 | 1 | 0 | 0 | 0 | 0 | 0 | ? | 0 | 0 | 0 | 0 | 0 | 0 | 0 | 0 | 0 | 0 | 0 | 0 | 0 | 2 | 0 | 0 | 0 | 0 | 0 | 0 | 0 | 0 | 0 | 0 | 0 | 0 | 0 | – | 0 | 0 | 1 | – | – | 0 | 1 | 0 | 0 | 0 | 0 | 0 | 0 |
| ? | 0 | 0 | ? | 0 | 0 | ? | ? | 0 | 0 | 0 | 0 | 0 | 0 | 0 | 0 | 0 | 0 | 0 | 0 | 0 | 0 | 0 | 0 | 0 | ? | ? | ? | ? | ? | ? | ? | ? | ? | ? | 0 | 0 | – | ? | ? | ? | – | – | ? | 1 | ? | ? | ? | ? | ? | ? |
| 0 | 0 | 0 | 1 | 0 | 0 | 0 | 0 | 0 | 0 | 1 | 1 | – | – | 0 | 0 | – | – | – | – | – | – | – | – | – | – | – | – | – | – | – | – | – | – | – | 0 | 0 | – | 0 | 0 | 1 | – | – | 0 | 1 | 0 | 0 | 0 | 0 | 0 | 0 |
| ? | ? | ? | ? | 1 | ? | ? | ? | 0 | 0 | 0 | 0 | 0 | ? | 0 | 0 | 0 | 0 | 0 | 0 | 0 | 0 | 0 | 0 | 0 | ? | ? | ? | ? | ? | ? | ? | ? | ? | ? | ? | ? | ? | ? | ? | ? | ? | ? | ? | ? | ? | ? | ? | ? | ? | ? |
| ? | ? | ? | ? | 1 | 1 | ? | 1 | 0 | 0 | 0 | 0 | 0 | ? | 0 | 0 | 2 | – | 0 | 0 | 0 | 1 | 0 | 0 | 0 | ? | ? | ? | ? | ? | ? | ? | ? | 0 | ? | ? | ? | ? | ? | ? | ? | ? | ? | ? | ? | ? | ? | ? | ? | ? | ? |
| ? | ? | ? | ? | 1 | 1 | ? | 1 | 0 | 0 | 0 | 0 | 0 | ? | 0 | 0 | 2 | – | 0 | 0 | 0 | 1 | 0 | 0 | 0 | 0 | 0 | 0 | 0 | 0 | 1 | 1 | 0 | 0 | ? | ? | ? | ? | ? | ? | ? | ? | ? | ? | ? | ? | ? | ? | ? | ? | ? |
| ? | ? | ? | ? | ? | ? | ? | ? | 0 | 0 | 0 | 0 | 0 | ? | 1 | 0 | 1 | 0 | 0 | 0 | 1 | 1 | 0 | 0 | 0 | 0 | 0 | 0 | 0 | 0 | 1 | 1 | 0 | 0 | ? | ? | ? | ? | ? | ? | ? | ? | ? | ? | ? | ? | ? | ? | ? | ? | ? |
| ? | ? | ? | ? | ? | ? | ? | ? | 0 | ? | 0 | 0 | 0 | ? | 1 | 0 | 0 | 0 | 0 | 0 | 1 | 1 | 0 | 0 | 0 | ? | ? | ? | ? | ? | ? | ? | ? | 0 | ? | ? | ? | ? | ? | ? | ? | ? | ? | ? | ? | ? | ? | ? | ? | ? | ? |
| ? | ? | ? | ? | ? | ? | ? | ? | 0 | 0 | 0 | 0 | 0 | ? | 0 | 1 | 0 | 0 | 0 | 1 | 1 | 1 | 0 | 0 | 0 | 0 | 0 | 1 | 0 | 0 | 1 | 1 | 0 | 0 | ? | ? | ? | ? | ? | ? | ? | ? | ? | ? | ? | ? | ? | ? | ? | ? | ? |
| ? | ? | ? | ? | ? | ? | ? | ? | 0 | ? | 0 | 0 | 0 | ? | 0 | 1 | 0 | 0 | 0 | 1 | 1 | 1 | 0 | 0 | 0 | ? | ? | ? | ? | ? | ? | ? | ? | ? | ? | ? | ? | ? | ? | ? | ? | ? | ? | ? | ? | ? | ? | ? | ? | ? | ? |
| ? | ? | ? | ? | ? | ? | ? | ? | 0 | ? | 0 | 0 | 0 | ? | 0 | 1 | 2 | 0 | 0 | 1 | 1 | 1 | 0 | 0 | 0 | ? | ? | ? | ? | ? | ? | ? | ? | ? | ? | ? | ? | ? | ? | ? | ? | ? | ? | ? | ? | ? | ? | ? | ? | ? | ? |
| ? | ? | ? | ? | ? | ? | ? | ? | 0 | ? | 0 | 0 | 0 | ? | 0 | 1 | 2 | 0 | 0 | 1 | 1 | 1 | 0 | 0 | 0 | ? | ? | ? | ? | ? | ? | ? | ? | ? | ? | ? | ? | ? | ? | ? | ? | ? | ? | ? | ? | ? | ? | ? | ? | ? | ? |
| ? | 0 | 0 | ? | ? | ? | ? | ? | 0 | 0 | 0 | 0 | 0 | 0 | 0 | 1 | 1 | 0 | 1 | 0 | 1 | 1 | 1 | 0 | 0 | 0 | 1 | 0 | 1 | 1 | 1 | 1 | 1 | 1 | 1 | 0 | 0 | 0 | 0 | 0 | 1 | ? | ? | 0 | 1 | 1 | 0 | 1 | 0 | 0 | 0 |
| 1 | 0 | 0 | ? | 1 | 0 | ? | 1 | 0 | 0 | 0 | 0 | 0 | 0 | 0 | 1 | 1 | 0 | 1 | 0 | 1 | 1 | 1 | 0 | 0 | 0 | 1 | 0 | 1 | 1 | 1 | 1 | 1 | 1 | 1 | 0 | 1 | ? | ? | ? | ? | 1 | 1 | 1 | 1 | 1 | 1 | 1 | 0 | 0 | 0 |
| ? | 0 | 0 | ? | 1 | 0 | ? | 1 | 0 | 0 | 0 | 0 | 0 | 0 | 0 | 1 | 1 | 0 | 1 | 1 | 1 | 1 | 0 | 0 | 0 | 0 | 1 | 0 | 1 | 1 | 1 | 1 | 1 | 1 | 1 | 0 | 0 | 0 | ? | ? | 1 | ? | ? | ? | 1 | ? | ? | ? | ? | ? | ? |
| ? | 0 | 0 | ? | ? | ? | ? | ? | 0 | 0 | 0 | 0 | 0 | 0 | 0 | 1 | 1 | 0 | 1 | 1 | 1 | 1 | 0 | 0 | 0 | 0 | 1 | 0 | 1 | 1 | 1 | 1 | 1 | 1 | 1 | 0 | 0 | 0 | 0 | 0 | 1 | ? | ? | ? | 1 | ? | ? | 1 | 0 | 0 | 0 |
| ? | 0 | 0 | ? | ? | ? | ? | ? | 0 | 0 | 0 | 0 | 0 | 0 | 0 | 1 | 1 | 0 | 1 | 1 | 1 | 1 | 0 | 0 | 0 | 0 | 1 | 0 | 1 | 1 | 1 | 1 | 1 | 1 | 1 | 0 | 0 | 0 | 0 | 0 | 1 | ? | ? | ? | 1 | ? | ? | 1 | 0 | 0 | 0 |
| ? | 0 | 0 | ? | 1 | ? | ? | 1 | 0 | 0 | 0 | 0 | 0 | 0 | 0 | 1 | 1 | 0 | 1 | 1 | 1 | 1 | 0 | 0 | 0 | ? | ? | ? | ? | ? | ? | ? | ? | ? | ? | 0 | 0 | ? | ? | ? | ? | ? | 1 | 0 | 1 | ? | 1 | 1 | 1 | 0 | 0 |
| ? | 0 | 0 | 0 | 1 | 0 | ? | 1 | 0 | 0 | 1 | 1 | 0 | 0 | 1 | 1 | 1 | 0 | 1 | 0 | 1 | 1 | 0 | 0 | 1 | ? | ? | ? | ? | ? | ? | ? | ? | ? | ? | 0 | 0 | ? | ? | ? | ? | ? | 0 | 1 | 0 | ? | 0 | ? | ? | ? | ? |
| 1 | 0 | 0 | 0 | 1 | 0 | 0 | 1 | 0 | 0 | 1 | 1 | 0 | 0 | 1 | 1 | 1 | 1 | 1 | 1 | 1 | 1 | 0 | 0 | 1 | ? | ? | ? | ? | ? | ? | ? | ? | ? | ? | 0 | 0 | ? | ? | ? | ? | 1 | 0 | 1 | 0 | 1 | 0 | 1 | 0 | 0 | 1 |
| 1 | 0 | 0 | 0 | 1 | 0 | 0 | 1 | 0 | 0 | 1 | 1 | 0 | 0 | 1 | 1 | 1 | 1 | 1 | 1 | 1 | 1 | 0 | 0 | 1 | ? | ? | ? | ? | ? | ? | ? | ? | ? | ? | 0 | 0 | ? | ? | ? | ? | ? | 0 | ? | 0 | ? | 0 | ? | ? | ? | ? |
| 1 | 0 | 0 | 0 | 1 | 0 | 0 | 1 | 0 | 0 | 1 | 1 | 0 | 0 | 1 | 1 | 2 | – | 1 | 1 | 1 | 1 | 0 | 0 | 1 | ? | ? | ? | ? | ? | ? | ? | ? | ? | ? | 0 | 0 | ? | ? | ? | ? | 1 | 0 | 1 | 0 | 1 | 0 | 1 | 0 | 0 | 1 |
| ? | 0 | 0 | 0 | 1 | 0 | ? | 1 | 0 | 0 | 1 | 1 | 0 | 0 | ? | ? | ? | 0 | ? | 0 | 1 | 1 | ? | ? | ? | ? | ? | ? | ? | ? | ? | ? | ? | ? | ? | 0 | 0 | 1 | ? | ? | ? | ? | ? | ? | ? | ? | ? | 1 | 0 | 0 | 0 |
| 1 | 0 | 0 | 0 | 1 | 0 | 0 | 1 | 1 | 1 | 2 | 2 | – | – | – | – | – | – | – | – | – | – | – | – | – | – | – | – | – | – | – | – | – | – | – | 0 | 0 | ? | ? | ? | ? | ? | ? | 0 | 1 | ? | ? | 1 | 0 | 0 | 0 |
| 1 | 0 | 0 | 0 | 0 | 0 | 1 | 1 | 0 | ? | 1 | 1 | ? | 1 | ? | ? | ? | ? | ? | ? | ? | ? | ? | ? | ? | ? | ? | ? | ? | ? | ? | ? | ? | ? | ? | 0 | 0 | ? | 0 | 0 | ? | ? | ? | 0 | 1 | ? | ? | 1 | 0 | 0 | 0 |
| 1 | 0 | 0 | 0 | 0 | 1 | 1 | 1 | 1 | 1 | 2 | 2 | – | – | – | – | – | – | – | – | – | – | – | – | – | – | – | – | – | – | – | – | – | – | – | 0 | 0 | ? | 0 | 0 | ? | ? | ? | 0 | 1 | ? | ? | 1 | 0 | 0 | 0 |
| 1 | 0 | 0 | 0 | 0 | 1 | ? | 0 | 1 | 1 | 2 | 2 | – | – | – | – | – | – | – | – | – | – | – | – | – | – | – | – | – | – | – | – | – | – | – | 0 | 0 | ? | ? | ? | ? | ? | ? | ? | ? | ? | ? | 1 | 0 | 0 | 0 |
| 1 | 0 | 0 | 0 | 0 | 1 | ? | 0 | 1 | 1 | 2 | 2 | – | – | – | – | – | – | – | – | – | – | – | – | – | – | – | – | – | – | – | – | – | – | – | 0 | 0 | ? | ? | ? | ? | ? | ? | ? | ? | ? | ? | 1 | 0 | 0 | 0 |
| ? | 0 | 0 | 0 | 0 | 1 | ? | 1 | 1 | 1 | 2 | 2 | – | – | – | – | – | – | – | – | – | – | – | – | – | – | – | – | – | – | – | – | – | – | – | 0 | 0 | ? | ? | ? | ? | ? | ? | ? | ? | ? | ? | 1 | 1 | 0 | 0 |
| 1 | 0 | 0 | 0 | 0 | 1 | 1 | 1 | 1 | 1 | 2 | 2 | – | – | – | – | – | – | – | – | – | – | – | – | – | – | – | – | – | – | – | – | – | – | – | 0 | 0 | 1 | 0 | 0 | 0 | 1 | 1 | 0 | 1 | 1 | 1 | 1 | 0 | 1 | 2 |
| 1 | 0 | 0 | 0 | 1 | 0 | 0 | 1 | 0 | 1 | 2 | 1 | 0 | 1 | – | – | – | – | – | – | – | – | – | – | – | 0 | 1 | 0 | 0 | 1 | 1 | 1 | 2 | – | 1 | 0 | 0 | 1 | 0 | 0 | 0 | 0 | 1 | 0 | 1 | 1 | 1 | 1 | 1 | 1 | 0 |
| 1 | 0 | 0 | 0 | 1 | 0 | 0 | 1 | 1 | 1 | 2– | 2 | – | – | – | – | – | – | – | – | – | – | – | – | – | – | – | – | – | – | – | – | – | – | – | 0 | 0 | 1 | 0 | 0 | 0 | 0 | 1 | 0 | 1 | 1 | 1 | 1 | 0 | 0 | 0 |
| 1 | 0 | 0 | 0 | 1 | 0 | 0 | 1 | 1 | 1 | 1 | 1 | – | – | – | – | – | – | – | – | – | – | – | – | – | 1 | – | – | – | 2 | 1 | 1 | 2 | – | – | 1 | 0 | 0 | 1 | 1 | 0 | 1 | 1 | 0 | 1 | 1 | 1 | 1 | 0 | 0 | 0 |
| 1 | 1 | 0 | 0 | 1 | 0 | 0 | 1 | 0 | 1 | 1 | 1 | 0 | 1 | 1 | 1 | 1 | 0 | 1 | 0 | 1 | 1 | 0 | 1 | 1 | 0 | 1 | 0 | 0 | 1 | 1 | 1 | 2 | – | 1 | 0 | 1 | 1 | 0 | 0 | 0 | 1 | 1 | 0 | 1 | 1 | 1 | 1 | 0 | 1 | 0 |
| 1 | 0 | 0 | 0 | 1 | 0 | 0 | 1 | 1 | 1 | 2 | 2 | – | – | – | – | – | – | – | – | – | – | – | – | – | – | – | – | – | – | – | – | – | – | – | 0 | 1 | 1 | 0 | 0 | 0 | 0 | 1 | 0 | 1 | 1 | 1 | 1 | 0 | 0 | 0 |
| 1 | 1 | 1 | 0 | 1 | 0 | 0 | 1 | 0 | 1 | 1 | 1 | 1 | 1 | 1 | 1 | 1 | 1 | 1 | 1 | 1 | 1 | 0 | 2 | 1 | 0 | 1 | 1 | 0 | 1 | 1 | 1 | 2 | – | 1 | 2 | 1 | 0 | 0 | 0 | 0 | 1 | 1 | 0 | 1 | 1 | 1 | 1 | 0 | 0 | 0 |
| 1 | 0 | 0 | 1 | 1 | 1 | 0 | 1 | 1 | 1 | 2 | 2 | – | – | – | – | – | – | – | – | – | – | – | – | – | – | – | – | – | – | – | – | – | – | – | 0 | 0 | 0 | 0 | 1 | 0 | 1 | 1 | 0 | 1 | 1 | 1 | 1 | 0 | 0 | 0 |
| 1 | 0 | 0 | 1 | 1 | 0 | 0 | 1 | 1 | 1 | 2 | 2 | – | – | – | – | – | – | – | – | – | – | – | – | – | – | – | – | – | – | – | – | – | – | – | 0 | 0 | 0 | 0 | 0 | 0 | 1 | 1 | 0 | 1 | 0 | 0 | 1 | 1 | 0 | 0 |
| 1 | 0 | 0 | 1 | 1 | 0 | 0 | 1 | 0 | 1 | 1 | 0 | 1 | 1 | 0 | 1 | – | – | – | – | – | – | – | – | – | 1 | – | – | – | 1 | 1 | 1 | 2 | – | 0 | 0 | 0 | 1 | 0 | 0 | 0 | 1 | 1 | 0 | 1 | 1 | 1 | 1 | 0 | 0 | 0 |
| 1 | 0 | 0 | 0 | 1 | 0 | 0 | 1 | 0 | 1 | 1 | 1 | – | – | – | – | – | – | – | – | – | – | – | – | – | 1 | – | – | – | 2 | – | – | 2 | – | – | 0 | 0 | 1 | 0 | 1 | 1 | 1 | 1 | 0 | 1 | 1 | 1 | 1 | 0 | 0 | 0 |
| 1 | 0 | 0 | 0 | 1 | 0 | 0 | 1 | 1 | 1 | 2 | 2 | – | – | – | – | – | – | – | – | – | – | – | – | – | – | – | – | – | – | – | – | – | – | – | 1 | 0 | 1 | 0 | 1 | 0 | 1 | 1 | 1 | 1 | 1 | 0 | 1 | 0 | 1 | 0 |
| 1 | 0 | 0 | 1 | 1 | 0 | 0 | 1 | 0 | 1 | 1 | 1 | 1 | 1 | 0 | 1 | – | – | – | – | – | – | – | – | – | 1 | – | – | – | 2 | – | – | 2 | – | 0 | 0 | 0 | 1 | 0 | 1 | 0 | 1 | 1 | 0 | 1 | 1 | 1 | 1 | 0 | 0 | 0 |
| 1 | 1 | 1 | 0 | 1 | 0 | 0 | 1 | 0 | 1 | 1 | 1 | 1 | 1 | 0 | 1 | 2 | 1 | 2 | – | – | – | – | 2 | 1 | 1 | – | – | – | 2 | – | – | 2 | – | 0 | 0 | 1 | 1 | 0 | 0 | 0 | 1 | 1 | 0 | 1 | 1 | 0 | 1 | 0 | 1 | 0 |
| 1 | 0 | 0 | 0 | 1 | 1 | 0 | 1 | 1 | 1 | 2 | 2 | – | – | – | – | – | – | – | – | – | – | – | – | – | – | – | – | – | – | – | – | – | – | – | 0 | 0 | 1 | 0 | 0 | 0 | 1 | 1 | 1 | 1 | 1 | 1 | 1 | 0 | 0 | 0 |
| 1 | 1 | 1 | 0 | 1 | 1 | 0 | 1 | 1 | 1 | 2 | 2 | – | – | – | – | – | – | – | – | – | – | – | – | – | – | – | – | – | – | – | – | – | – | – | 0 | 1 | ? | 0 | 0 | ? | ? | 1 | 0 | 1 | ? | ? | 1 | 0 | 0 | 0 |
| 1 | 0 | 0 | ? | 1 | 0 | 0 | 1 | 1 | 1 | 2 | 2 | – | – | – | – | – | – | – | – | – | – | – | – | – | – | – | – | – | – | – | – | – | – | – | 0 | 0 | ? | ? | ? | ? | ? | ? | 0 | ? | ? | ? | 1 | 0 | 0 | 0 |
| 1 | 0 | 0 | ? | 1 | 0 | 0 | 1 | 1 | 1 | 2 | 2 | – | – | – | – | – | – | – | – | – | – | – | – | – | – | – | – | – | – | – | – | – | – | – | 1 | 1 | ? | ? | ? | ? | ? | ? | 0 | ? | ? | ? | 1 | 0 | 0 | 0 |
| 1 | ? | ? | ? | ? | ? | ? | ? | 0 | 1 | 1 | 1 | 1 | 1 | 1 | 1 | ? | ? | ? | ? | ? | ? | ? | ? | 1 | ? | ? | ? | ? | ? | ? | ? | ? | ? | ? | 2 | 1 | ? | ? | ? | ? | ? | ? | 0 | 1 | 1 | 1 | 1 | 0 | 0 | 0 |
| 1 | 0 | 0 | 1 | 1 | 0 | ? | 1 | 1 | 1 | 2 | 2 | – | – | – | – | – | – | – | – | – | – | – | – | – | – | – | – | – | – | – | – | – | – | – | 1 | 0 | ? | ? | ? | ? | ? | ? | 0 | 1 | ? | ? | 1 | 0 | 0 | 0 |

**Table S3.** The list of described Phasmatodea fossils.

| **Family** | **Genus** | **species** | **Epoch and Age** | **Country** |
| --- | --- | --- | --- | --- |
| Permophasmatidae | *Permophasma* | *P. kovalevi* Gorochov, 1992 | 260–254 Ma  (Permian) | Mongolia  (South Gobi Aymag) |
|  | *Isadyphasma* | *I. suchonae* Gorochov, 2013 | 260–254 Ma  (Permian) | Russian Federation  (Vologda Regin) |
|  |  | *I. deminutum* Gorochov, 2013 | 260–254 Ma  (Permian) | Russian Federation  (Vologda Regin) |
|  |  | *I. bashkuevi* Gorochov, 2013 | 260–254 Ma  (Permian) | Russian Federation  (Vologda Regin) |
|  | *Arachnophasma* | *A. scurra* Aristov & Rasnitsyn, 2015 | 279–272 Ma (Permian) | Russian Federation (Tshekarda) |
| Prochresmodidae | *Palaeochresmoda* | *P. grauvogeli* Nel et al., 2004 | 247–242 Ma  (Late Triassic) | France  (Moselle) |
|  | *Prochresmoda* | *P. brevipoda* Sharov, 1968 | 237–228 Ma  (Late Triassic) | Kyrgyzstan  (Osh Region) |
|  |  | *P. longipoda* Sharov, 1968 | 237–228 Ma  (Late Triassic) | Kyrgyzstan  (Osh Region) |
|  |  | *P. media* Gorochov, 1994 | 237–228 Ma  (Late Triassic) | Kyrgyzstan  (Osh Region) |
|  |  | *P. minuta* Gorochov, 1994 | 237–228 Ma  (Late Triassic) | Kyrgyzstan  (Osh Region) |
|  |  | *P. parva* Gorochov, 1994 | 237–228 Ma  (Late Triassic) | Kyrgyzstan  (Osh Region) |
|  | *Triassophasma* | *T. intermedium* Gorochov, 1994 | 237–228 Ma  (Late Triassic) | Kyrgyzstan  (Osh Region) |
|  |  | *T. minutissimum* Gorochov, 1994 | 237–228 Ma  (Late Triassic) | Kyrgyzstan  (Osh Region) |
|  |  | *T. pusillum* Gorochov, 1994 | 237–228 Ma  (Late Triassic) | Kyrgyzstan  (Osh Region) |
| Xiphopteridae | *Xiphopterum* | *X. curvatum* Sharov, 1968 | 237–228 Ma  (Late Triassic) | Kyrgyzstan  (Osh Region) |
|  |  | *X. sharovi* Gorochov, 1994 | 237–228 Ma  (Late Triassic) | Kyrgyzstan  (Osh Region) |
| Aeroplanidae | *Aeroplana* | *A. mirabilis* Tillyard, 1918 | 228–209 Ma  (Late Triassic) | Australia  (Queensland) |
|  | *Sharovoplana* | *S. affinis* Sharov, 1968 | 237–228 Ma  (Late Triassic) | Kyrgyzstan  (Osh Region) |
|  |  | *S. parallelica* Goroshov, 1994 | 237–228 Ma  (Late Triassic) | Kyrgyzstan  (Osh Region) |
| Aerophasmatidae | *Cretophasma* | *C. raggei* Sharov, 1968 | 93.9–89.8 Ma  (Late Cretaceous) | Kazakhstan  (Kzyi-Orda Oblast) |
|  |  | *C. araripensis* Martins-Neto, 1989 | 115–110 Ma  (Early Cretaceous) | Brazil  (Ceará) |
|  | *Aerophasma* | *A. prynadai* Martynov, 1928 | 166–157 Ma  (Middle Jurassic) | Kazakhstan  (Chimkent Oblast) |
|  | *Baissophasma* | *B. pilosum* Gorochov, 1993 | 125–113 Ma  (Early Cretaceous) | Russian Federation (Transbaikalia) |
|  |  | *B. simile* Gorochov, 1993 | 125–113 Ma  (Early Cretaceous) | Russian Federation  (Transbaikalia) |
|  | *Chresmodella* | *C. convoluta* Bode, 1953 | 183–182 Ma  (Early Jurassic) | Germany  (Neidersachsen) |
|  |  | *C. fissa* Bode, 1953 | 183–182 Ma  (Early Jurassic) | Germany  (Neidersachsen) |
|  |  | *C. lacerata* Bode, 1953 | 183–182 Ma  (Early Jurassic) | Germany  (Neidersachsen) |
|  |  | *C. integra* Bode, 1953 | 183–182 Ma  (Early Jurassic) | Germany  (Neidersachsen) |
|  |  | *C. culminecontracta* Bode, 1953 | 183–182 Ma  (Early Jurassic) | Germany  (Neidersachsen) |
|  | *Durnovaria* | *D. parallela* Whalley, 1985 | 183–182 Ma  (Early Jurassic) | UK  (England, Dorset) |
|  | *Jurophasma* | *J. mistshenkoi* Gorochov, 1993 | 166–157 Ma  (Middle Jurassic) | Kazakhstan  (Chimkent Oblast) |
|  | *Schesslitziella* | *S. haupti* Kuhn, 1952 | 183–182 Ma  (Early Jurassic) | Germany  (Bavaria) |
| Susumaniidae | *Aethephasma* | *A. megista* Ren, 1997 | ~125 Ma  (Early Cretaceous) | China  (Pingquan, Hebei) |
|  | *Hagiphasma* | *H. paradoxa* Ren, 1997 | ~125 Ma  (Early Cretaceous) | China  (Beipiao, Liaoning) |
|  | *Orephasma* | *O. eumorpha* Ren, 1997 | ~125 Ma  (Early Cretaceous) | China  (Pingquan, Hebei) |
|  | *Renphasma* | *R. sinica* Nel & Delfosse, 2011 | ~125 Ma  (Early Cretaceous) | China  (Beipiao, Liaoning) |
|  | *Adjacivena* | *A. rasnitsyni* Shang, Béthoux & Ren, 2011 | ~165 Ma  (Middle Jurassic) | China (Ningcheng, Inner Mongolia) |
|  |  | *A.* *grossa* Yang, Engel, Ren & Gao, 2022 | ~165 Ma  (Middle Jurassic) | China (Ningcheng, Inner Mongolia) |
|  | *Aclistophasma* | *A. echinulatum* Yang, Engel & Gao, 2020 | ~165 Ma  (Middle Jurassic) | China (Ningcheng, Inner Mongolia) |
|  | *Coniphasma* | *C. rosenkrantzi* Birket-Smith, 1981 | 89.8–86.3 Ma  (Late Cretaceous) | Greenland |
|  | *Cretophasmomima* | *C. melanogramma* Wang, Béthoux & Ren, 2014 | ~125 Ma  (Early Cretaceous) | China (Ningcheng, Inner Mongolia) |
|  |  | *C. vitimica* Kuzmina, 1985 | 125–113 Ma  (Early Cretaceous) | Russian Federation  (Transbaikalia) |
|  |  | *C. burjatica* Gorochov, 1988 | 125–113 Ma  (Early Cretaceous) | Russian Federation  (Transbaikalia) |
|  |  | *C. clara* Gorochov, 1988 | 83.5–70.6 Ma  (Late Cretaceous) | Russian Federation  (Magadan) |
|  |  | *C. arkagalica* Gorochov, 1988 | 83.5–70.6 Ma  (Late Cretaceous) | Russian Federation  (Magadan) |
|  |  | *C. traceyae* Xu et al., 2020 | 145–125 Ma  (Early Cretaceous) | UK (England, Ockley village, Surrey County) |
|  | *Cretophasmomimoides* | *C. reductus* Gorochov, 1988 | 125–113 Ma  (Early Cretaceous) | Mongolia  (Uverkhangai Aymag) |
|  | *Liutiaogoucuna* | *L. arachnoidea* Xu et al., 2020 | ~125 Ma  (Early Cretaceous) | China (Ningcheng, Inner Mongolia) |
|  | *Eosusumania* | *E. fusca* Gorochov, 1988 | 93.9–89.8 Ma  (Late Cretaceous) | Russian Federation  (Khabarovski) |
|  |  | *E. reticulata* Kuzmina, 1985 | 125–113 Ma  (Early Cretaceous) | Russian Federation  (Transbaikalia) |
|  |  | *E. ornata* Kuzmina, 1985 | 125–113 Ma  (Early Cretaceous) | Russia  (Transbaikalia) |
|  |  | *E. maculata* Kuzmina, 1985 | 125–113 Ma  (Early Cretaceous) | Russian Federation  (Transbaikalia) |
|  |  | *E. lata* Kuzmina, 1985 | 125–113 Ma  (Early Cretaceous) | Russian Federation  (Transbaikalia) |
|  | *Palaeopteron* | *P. complexus* Rice, 1969 | 101–94 Ma  (Late Cretaceous) | Canada  (Labrador) |
|  | *Paraphasmomimella* | *P. longa* Kuzmina, 1985 | 125–113 Ma  (Early Cretaceous) | Russian Federation  (Transbaikalia) |
|  |  | *P. chetanica* Gorochov, 1988 | 93.9–89.8 Ma  (Late Cretaceous) | Russian Federation  (Khabarovski) |
|  | *Phasmomimella* | *P. araripensis* Martins-Neto, 1991 | 115–110 Ma  (Early Cretaceous) | Brazil  (Ceará) |
|  |  | *P. antiqua* Kuzmina, 1985 | 125–113 Ma  (Early Cretaceous) | Russian Federation  (Transbaikalia) |
|  |  | *P. paskapoensis* Kevan & Wighton, 1981 | 61.7–56.8 Ma  (Paleocene) | Canada  (Alberta) |
|  | *Phasmomimoides* | *P. lineatus* Sharov, 1968 | 166–157 Ma  (Middle Jurassic) | Kazakhstan  (Chimkent Oblast) |
|  |  | *P. baissiense* Sharov, 1968 | 125–113 Ma  (Early Cretaceous) | Russian Federation  (Transbaikalia) |
|  |  | *P. reticulatus* Kuzmina, 1985 | 125–113 Ma  (Early Cretaceous) | Russian Federation  (Transbaikalia) |
|  |  | *P. latus* Kuzmina, 1985 | 125–113 Ma  (Early Cretaceous) | Russian Federation  (Transbaikalia) |
|  |  | *P. ornatus* Kuzmina, 1985 | 125–113 Ma  (Early Cretaceous) | Russian Federation  (Transbaikalia) |
|  |  | *P. maculatus* Kuzmina, 1985 | 125–113 Ma  (Early Cretaceous) | Russian Federation  (Transbaikalia) |
|  |  | *P. minutus* Gorochov, 2000 | 166–157 Ma  (Middle Jurassic) | Kazakhstan  (Chimkent Oblast) |
|  | *Phasmomimula* | *P. enigma* Kevan & Wighton, 1981 | 61.7–56.8 Ma  (Paleocene) | Canada  (Alberta) |
|  | *Promastacoides* | *P. albertae* Kevan & Wighton, 1981 | 61.7–56.8 Ma  (Paleocene) | Canada  (Alberta) |
|  | *Prosusumania* | *P. semenica* Gorochov, 1988 | 125–122 Ma  (Early Cretaceous) | Russian Federation  (Pad Semen) |
|  | *Susumania* | *S. flexuosa* Gorochov, 1988 | 83.5–70.6 Ma  (Late Cretaceous) | Russian Federation  (Magadan) |
|  | *Eoprephasma* | *E. hichensi* Archibald & Bradler, 2015 | ~49 Ma  (Eocene) | Canada  (British Columbia) |
| Pterophasmatidae | *Pterophasma* | *P. erromera* Yang, Shih, Ren & Gao, 2019 | ~ 99 Ma  (Late Cretaceous) | Kachin amber |
|  | *Leptophasma* | *L. physematosa* Yang, Shih, Ren & Gao, 2019 | ~ 99 Ma  (Late Cretaceous) | Kachin amber |
|  | *Meniscophasma* | *M. erythrosticta* Yang, Shih, Ren & Gao, 2019 | ~ 99 Ma  (Late Cretaceous) | Kachin amber |
|  | *Ambrotophasma* | *A. vetulum* Yang, Engel, Ren & Gao, 2022 | ~165 Ma  (Middle Jurassic) | China (Ningcheng, Inner Mongolia) |
| Archipseudophasmatidae | *Pseudoperla* | *P. leptoclada* Chen, Shih & Ren, 2017 | ~ 99 Ma  (Late Cretaceous) | Kachin amber |
|  |  | *P. scapiforma* Chen, Shih & Ren, 2017 | ~ 99 Ma  (Late Cretaceous) | Kachin amber |
|  |  | *P. sleipnir* Zompro, 2001 | 38–33.9 Ma  (Eocene) | Baltic amber |
|  |  | *P. lineata* Pictet & Berendt, 1854 | 38–33.9 Ma  (Eocene) | Baltic amber |
|  |  | *P. gracilipes* Pictet & Berendt, 1854 | 38–33.9 Ma  (Eocene) | Baltic amber |
|  | *Archipseudophasma* | *A. phoenix* Zompro, 2001 | 38–33.9 Ma  (Eocene) | Baltic amber |
|  | *Sucinophasma* | *S. blattodeophila* Zompro, 2004 | 38–33.9 Ma  (Eocene) | Baltic amber |
|  | *Dvergrphasma* | *D. fafnir* Zompro, 2005 | 38–33.9 Ma  (Eocene) | Baltic amber |
|  | *Balticophasma* | *B. lineata* Germar & Berendt, 1856 | 38–33.9 Ma  (Eocene) | Baltic amber |
|  | *Electrobaculum* | *E. gracile* Sharov, 1968 | 38–33.9 Ma  (Eocene) | Baltic amber |
| Timematodea | *Tumefactipes* | *T. prolongates* Chen, Shih, Ren & Gao, 2018 | ~ 99 Ma  (Late Cretaceous) | Kachin amber |
|  | *Granosicorpes* | *G. lirates* Chen, Shih, Ren & Gao, 2018 | ~ 99 Ma  (Late Cretaceous) | Kachin amber |
|  | *Breviala* | *B. cretacea* Yang, Engel, Shih & Gao, 2023 (herein) | ~ 99 Ma  (Late Cretaceous) | Kachin amber |
|  | *Electroclavella* | *E. genuine* Yang, Engel, Shih & Gao, 2023 (herein) | ~ 99 Ma  (Late Cretaceous) | Kachin amber |
|  | *Electrotimema* | *E. carstengroehni* Zompro, 2005 | 38–33.9 Ma  (Eocene) | Baltic amber |
| Euphasmatodea | *Agathemera* | *A. reclusa* Scudder, 1890 | 37.2–33.9 Ma  (Eocene) | U.S.A.  (Colorado) |
|  | *Calcibacunculus* | *C. tenuis* Pierce, 1951 | 5.33–2.59 Ma  (Pliocene) | U.S.A.  (Arizona) |
|  | *Clonistria* | *C. dominicana* Poinar, 2011 | 20.4–13.8 Ma  (Miocene) | Dominican Republic |
|  | *Echinosomiscus* | *E. primoticus* Engel & Wang, 2016 | ~ 99 Ma  (Late Cretaceous) | Kachin amber |
|  | *Eophyllium* | *E. messelensis* Wedmann, Bradler & Rust, 2007 | ~ 47 Ma  (Eocene) | Germany  (Hessen) |
|  | *Elasmophasma* | *E. stictum* Chen, Shih, Gao & Ren, 2018 | ~ 99 Ma  (Late Cretaceous) | Kachin amber |
|  |  | *E.* *longitubus* Yang, Engel, Ren & Gao, 2022 | ~ 99 Ma  (Late Cretaceous) | Kachin amber |
|  | *Electrobaculum* | *E. gracile* Sharov, 1968 | 38–33.9 Ma  (Eocene) | Baltic amber |
|  | *Rhabdophasma* | *R.* *arboreum* Yang, Engel, Ren & Gao, 2022 | ~ 99 Ma  (Late Cretaceous) | Kachin amber |
|  | *Tanaophasma* | *T.* *applanatum* Yang, Engel, Ren & Gao, 2022 | ~ 99 Ma  (Late Cretaceous) | Kachin amber |
|  | *Araripephasma* | *A. reliquum* Ghirotto, Crispino & Neves, 2022 | 115–110 Ma  (Early Cretaceous) | Brazil  (Ceará) |
